# Exploration of the structural and functional diversity in the metamorphic RfaH subfamily

**DOI:** 10.64898/2026.03.16.712203

**Published:** 2026-03-18

**Authors:** Cyndi Tabilo-Agurto, Bastián González-Bustos, Javiera Reyes, Bing Wang, Damaris Palomera, Verónica Del Río-Pinilla, Camila Neira-Mahuzier, Valentina Vera-Sandoval, Irina Artsimovitch, Pablo Galaz-Davison, César A. Ramírez-Sarmiento

**Affiliations:** 1 Institute for Biological and Medical Engineering, Schools of Engineering, Medicine and Biological Sciences, Pontificia Universidad Católica de Chile, Santiago 7820436, Chile.; 2 ANID – Millennium Science Initiative Program, Millennium Institute for Integrative Biology (iBio), Santiago 833150, Chile.; 3 Department of Microbiology and Center for RNA Biology, The Ohio State University, Columbus, OH 43210, USA.; 4 Center for Bioinformatics, Simulation and Modeling, School of Engineering, Universidad de Talca, Talca 3460000, Chile.

**Keywords:** AlphaFold2, Metamorphic Proteins, RfaH, Transcription, Translation

## Abstract

RfaH, a member of the universally conserved NusG/Spt5 family, activates transcription and translation of virulence genes by bridging the transcribing RNA polymerase to the translating ribosome. Unlike its monomorphic, constitutively active paralog NusG, RfaH fold-switches from an autoinhibited state, wherein its C-terminal domain forms an α-helical hairpin bound to the N-terminal domain, into an active NusG-like state through C-terminal domain dissociation and refolding into a β-barrel. RfaH has been proposed to evolve from NusG via gene duplication by first activating genes adjacent to its production site, before developing autoinhibition to selectively control distant genes. Using AlphaFold2, we predicted the structures of thousands of RfaH homologs and identified a subset (~14%) that appear predominantly folded in the active state. *In vivo* assays confirmed that these putative monomorphic homologs exhibit constitutive activity comparable to known RfaH mutants. Phylogenetic and genomic analyses revealed that these monomorphic proteins form a distinct clade and are preferentially located within or next to long virulence-associated operons. Together, these results further support a stepwise evolutionary model of RfaH specialization through structural transformation.

## INTRODUCTION

Metamorphic proteins are characterized by their reversible fold-switch between two disparate, yet stable native states encoded by a single amino acid sequence, necessary for regulating or encoding different biological functions (Porter et al, 2024). These proteins have been discovered by solving their three-dimensional structures under different contexts, such as sequence truncations (Bullough et al, 1994; Burmann et al, 2012), residue substitutions that stabilize their different folds (Chang et al, 2015; Cai et al, 2020) or changes in environmental conditions (Littler et al, 2004; López-Pelegrín et al, 2014), with rare cases in which both native states are observed in a single experimental structure (Mapelli et al, 2007).

Over 100 proteins across all kingdoms of life have been described to switch folds (Porter & Looger, 2018), with recent discoveries in viral proteins suggesting that these numbers will increase (Cai et al, 2020; Arnold et al, 2026). In fact, bioinformatic analysis of folding cooperativity and ambiguous secondary structure predictions from sequence information suggest that ~4% of the proteins available in the PDB may switch folds (Porter & Looger, 2018).

While computational and experimental biophysics have shed light on the fold-switching mechanisms of several metamorphic proteins, such as *Escherichia coli* RfaH (Zuber et al, 2019; González-Higueras et al, 2024; Tabilo-Agurto et al, 2025; Cai et al, 2025), cyanobacterial KaiB (Chang et al, 2015; Rivera et al, 2022), and human XCL1 (Tuinstra et al, 2008; Camilloni & Sutto, 2009; Tyler et al, 2011), less is known about the evolutionary emergence of these proteins in nature. To date, only the emergence of XCL1 has been thoroughly explored by ancestral sequence reconstruction and nuclear magnetic resonance of these ancestors, suggesting that this protein evolved from an ancestor with a monomeric chemokine fold into a novel dimeric native state (Dishman et al, 2021). Overall, the consensus is that most, if not all, metamorphic proteins evolved by encoding an alternative native state within monomorphic protein families with a canonical fold (Dishman & Volkman, 2023; Porter et al, 2024).

The quintessential metamorphic virulence factor RfaH (Artsimovitch & Ramírez-Sarmiento, 2022) is another well-studied protein in terms of its evolution, with a concrete hypothesis of its emergence within the universally conserved NusG/Spt5 family of transcription elongation factors (Tomar & Artsimovitch, 2013). RfaH arose through gene duplication of NusG (Bailey et al, 1997), a protein that in *E. coli* is associated with RNA polymerase (RNAP) transcribing almost all genes (Mooney et al, 2009a). NusG is a monomorphic protein composed of two domains, the NusG N-terminal (NGN) domain (NTD), which binds to RNAP in transcription elongation complexes (TEC), and the C-terminal Kyrpides, Ouzounis, Woese (KOW) domain (CTD) folded as a β-barrel (βCTD) that binds to either Rho or the ribosome (Mooney et al, 2009b). NusG enables RNAP to monitor the RNA quality and cellular conditions by either promoting early transcription termination of foreign, antisense, or damaged RNAs by Rho (Cardinale et al, 2008; Peters et al, 2012), or by supporting transcription-translation coupling (Wang et al, 2020b; Webster et al, 2020).

In contrast, the *E. coli* paralog RfaH, with only 21% sequence identity to NusG, is a metamorphic protein. In its resting, autoinhibited state, the RfaH-CTD is folded as an α-helical hairpin (αCTD) tightly bound to the RNAP-binding site on the NTD (Burmann et al, 2012; Zuber et al, 2018). Despite a 100-fold cellular excess of NusG (Schmidt et al, 2016), RfaH excludes NusG from RNAP (Belogurov et al, 2009) due to its 10-fold higher affinity (Kang et al, 2018). In *Enterobacteriaceae*, RfaH dramatically activates expression of several long horizontally-acquired operons related to bacterial virulence (capsule, cell wall, toxins, adhesins, pilus and lipopolysaccharide biosynthesis [LPS]); these operons frequently lack ribosomal binding sites (RBS) and are silenced by the joint action of Rho and nucleoid-associated proteins (Hustmyer et al, 2022; Wang et al, 2022). RfaH is recruited to RNAP at a specific 12-nt sequence known as *operon polarity suppressor* (*ops*) (Bailey et al, 1997) that forms a short hairpin in the non-template DNA strand on the surface of a paused TEC (Artsimovitch & Landick, 2002; Zuber et al, 2018). The RfaH interactions with *ops* and RNAP triggers activation of RfaH by interdomain dissociation and fold-switching of its αCTD into the canonical βCTD of NusG (Burmann et al, 2012; Zuber et al, 2024). Importantly, once RfaH unbinds from the TEC, it folds back into its autoinhibited state (Zuber et al, 2019). While RfaH-CTD does not bind Rho (Lawson et al, 2018), it interacts with the ribosomal protein S10 to recruit the ribosome to RBS-less mRNAs (Burmann et al, 2012) and may subsequently couple transcription and translation (Molodtsov et al, 2024). By simultaneously excluding NusG and anchoring the ribosome, RfaH ensures the expression of distal genes within long *ops*-containing virulence operons.

It has been hypothesized that after *nusG* gene duplication, monomorphic ancestral RfaH with the canonical NusG fold (βRfaH) first lost their binding to Rho and their solubility, while gaining higher affinity for RNAP to regulate genes closely linked to its production site (i.e., in *cis*), impeding displacement by the more abundant NusG protein (Belogurov et al, 2009; Wang et al, 2020a). Then, RfaH would have evolved to gain DNA sequence specificity and solubility by folding into the autoinhibited state (αRfaH) to enable its action in *trans* over operons far from the genomic *loci* encoding RfaH (Belogurov et al, 2009; Wang et al, 2020a). However, there is neither direct or indirect evidence of extant, functional monomorphic βRfaH proteins, nor of their in-*cis* regulation.

Deep learning (DL) protein structure prediction methods such as AlphaFold2 (AF2) only predict one of the two native states of fold-switching proteins (Chakravarty & Porter, 2022; Chakravarty et al, 2024) However, different results are obtained when reducing the number of homologous protein sequences in the multiple sequence alignment (MSA) generated as input during the process (Wayment-Steele et al, 2024; Guan et al, 2024; Lee et al, 2025). Nevertheless, previous works have shown that AF2 in its default settings predicts the autoinhibited state for *E. coli* RfaH and for the distant homolog from *Vibrio cholerae*, which only shares 43% sequence identity (Artsimovitch & Ramírez-Sarmiento, 2022).

Based on the assumption that predicting the autoinhibited state is indicative of their metamorphic behavior, one can hypothesize that AF2 could be used to predict fold-switching RfaH orthologs. Conversely, predicting the canonical NusG-like state, which has been achieved using evolutionary-based and physics-based mutations of interdomain CTD residues in *E. coli* RfaH – some of which have been experimentally validated to be constitutively active in previous works (Shi et al, 2017; Freiberger et al, 2023; Tabilo-Agurto et al, 2025) – would suggest potential monomorphic candidates. This opens the door to explore the evolutionary hypothesis of the structural and functional emergence of the RfaH fold-switch within the NusG/Spt5 family.

**Figure 1:**
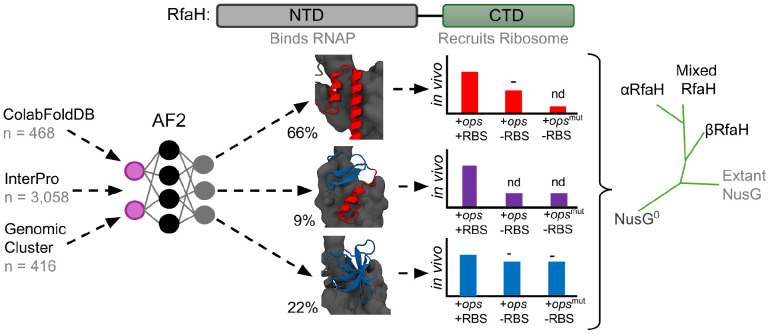
AF2-, activity-, and phylogeny-guided classification of the structural and functional diversity of extant RfaH homologs. (**Top**) Architecture of RfaH displaying the NTD and the fold-switching CTD connected by a linker. (**Bottom**) Scheme of the computational and experimental approach to explore the structural and functional diversity of extant RfaH proteins. The structures of >3,900 sequences from different databases were predicted using AF2 in ColabFold, and classified based on the secondary structure content of their CTD into either αCTD, mixed α/β CTD, or βCTD folds. Three representative RfaH homologs from the Genomic Cluster predicted in each state were subjected to RfaH-dependent *in vivo* transcription-translation assays to explore the effects of the *ops* signal and the RBS on their activity using a luminescence reporter. Lastly, a maximum likelihood phylogenetic analysis grouped these different extant proteins in separate clades for metamorphic αRfaH, monomorphic βRfaH, and RfaH with mixed α/β CTD, matching their structural and functional diversity. Nd = not detectable (similar to luminescence with condition lacking RfaH expression)

Based on these precedents, and also on recent discussions that fold-switching proteins are proof of protein folds existing in a continuum of states (Tian & Best, 2020; Porter, 2023; Kulkarni et al, 2025), we used ColabFold (Mirdita et al, 2022), a cloud-based implementation of AF2 (Jumper et al, 2021), to perform a large-scale protein structure prediction of RfaH homologs with different levels of sequence identity against *E. coli* RfaH from several sequence databases. While ~66% of the ~20,000 RfaH structures were predicted to present an αCTD, ~14% of the RfaH homologs (22% of the predicted structures) are mainly predicted in the βRfaH fold, suggesting they constitute potential monomorphic RfaH proteins. Moreover, 9% of the total predicted structures exhibited structures with mixed α/β secondary structure within their CTDs. These structures were observed in targeted molecular dynamics (TMD) (Schlitter et al, 1994) simulations of the *E. coli* RfaH fold-switch and their structures were assessed as reliable by the AF2-based structure scoring framework AF2Rank (Roney & Ovchinnikov, 2022), suggesting that they may represent evolutionary and structural intermediates of RfaH fold-switching (Figure 1).

Further, we selected nine RfaH homologs from a database of RfaH sequences with known genomic origin, whose predicted structures exhibited either αCTD, βCTD or mixed α/β CTD, for experimental testing using *in vivo* translation assays. Proteins with mixed α/β CTD fold did not display RfaH-dependent transcription-translation coupling, whereas monomorphic βRfaH proteins behaved similarly to the constitutively active E48A RfaH variant that does not require the *ops* for its activity (Burmann et al, 2012). Lastly, phylogenetic inference and genomic analysis of RfaH homologs and their predicted structures revealed that monomorphic βRfaH proteins cluster into a defined clade and are located next to pathogenicity-related long operons in their corresponding genomes, in contrast to metamorphic αRfaH homologs who operate in *trans*. Together, these results provide further evidence that RfaH evolved from monomorphic, NusG-like ancestors that acted locally near their production sites, a lineage that appears to remain extant in some species (Figure 1).

## RESULTS

### AF2 predicts multiple conformations for different RfaH homologs

Amino acid sequences for *E. coli* RfaH homologs were retrieved from ColabFold database (ColabFoldDB) (Mirdita et al, 2022), corresponding to 468 sequences after sequence filtering (see Methods); InterPro (Blum et al, 2025) (family entry IPR010215) with 3,058 sequences, and a previously reported list of 416 RfaH sequences (Cluster 1) curated by hidden Markov models (HMM) with known genomic context that were classified by Markov clustering and include *E. coli* RfaH (Genomic Cluster, hereafter) (Wang et al, 2020a).

These 3,942 sequences were used as input for DL-guided protein structure prediction using AF2 with the default ColabFold protocol, their TM-score (Zhang & Skolnick, 2004) (a normalized structure superimposition quality metric) and the RMSD against the αCTD and βCTD states from *E. coli* RfaH was assigned using TM-align (Zhang & Skolnick, 2005) and the secondary structure of their CTDs with STRIDE (Frishman & Argos, 1995) (see Methods). We employed this analysis to determine secondary structure thresholds that enabled us to distinguish between metamorphic RfaH (αRfaH), monomorphic RfaH (βRfaH) or RfaH with mixed α/β secondary structure in the CTD (Supplementary Figure S1). Predictions were also made using only the isolated CTDs of the proteins from each database, as experimental deletion of the NTD (Burmann et al, 2012) and ColabFold predictions (Artsimovitch & Ramírez-Sarmiento, 2022) have shown that the isolated CTD folds into the βCTD, and thus allow to test whether potential metamorphic proteins indeed switch folds (Supplementary Figure S2).

**Figure 2:**
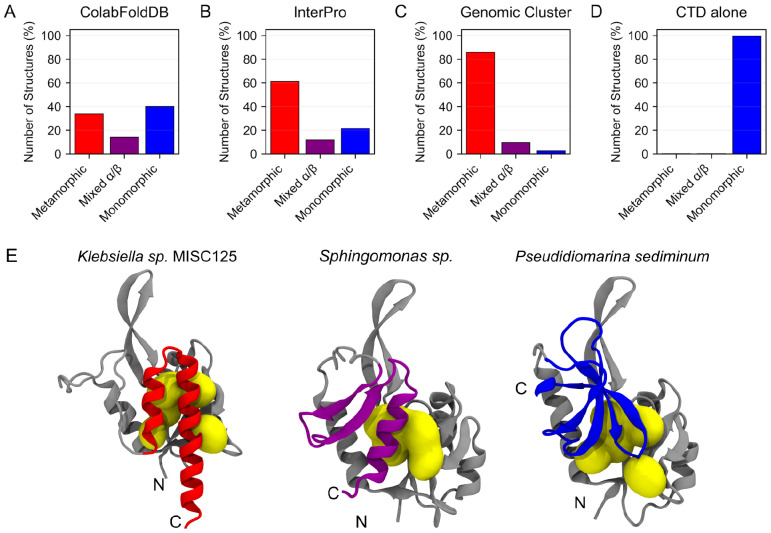
Distribution of predicted structures of extant RfaH homologs with αCTD, βCTD or mixed secondary structure from the ColabFoldDB (**A**), InterPro (**B**) and Genomic Cluster (**C**) databases. The secondary structure associated with the CTD region was ascertained using STRIDE and the CTD region was selected based on a sequence alignment to *E. coli* RfaH as reference. About 66% of the predicted structures of full-length RfaH homologs from ColabFoldDB (**A**) and InterPro (**B**) exhibit an αCTD, while ~22% are predicted with a βCTD and ~9% are predicted to have mixed α/β secondary structure. Most of the RfaH sequences from the Genomic Cluster (**C**) are predicted to fold into the αCTD, and when the isolated CTD from these sequences is used as input for protein structure prediction (**D**), they fold into the canonical βCTD of the NusG family. (**E**) Cartoon representations of representative RfaH homologs from the Genomic Cluster database exhibiting an αCTD (red), βCTD (blue) or mixed secondary structure (purple). The structurally conserved NTD is shown in gray, and interdomain contacts are shown in yellow.

Analysis of the 19,710 generated structures showed that AF2 predicted the autoinhibited state of RfaH in sequences from all three databases, although with different propensities (Figure 2 and Supplementary Figure 2). For the predicted structures from ColabFoldDB, which includes some representatives that are more distant to *E. coli* RfaH (mean sequence identity to *E. coli* RfaH = 31.5%), 33% of all 5 structures generated per sequence were predicted to fold as αRfaH, whereas 40% of the predicted structures had a βCTD fold resembling that of NusG (Figure 2A and Supplementary Figure 2A and 2C). These βRfaH structure predictions strongly suggest that these homologs might be monomorphic. Also, ~14% of the ColabFoldDB predicted structures of full-length RfaH homologs exhibited a CTD with mixed α/β secondary structure (Figure 2A and 2E and Supplementary Figure 2E). It is worth noting that about 12% of the predicted structures from ColabFoldDB were uncategorized due to their lack of secondary structure (data not shown).

The fraction of predicted structures with mixed α/β secondary structure is similar to ColabFoldDB in the case of the InterPro database, with 12% of the predicted structures exhibiting these patterns (Figure 2B and Supplementary Figure 3B), suggesting that the prediction of a mixed α/β CTD for some RfaH homologs is not rare for AF2. The predicted structures of the InterPro sequences also show an important increase from 34% structures predicted from ColabFoldDB in the αRfaH fold to 61% in InterPro, along with a decrease in the number of structures with a βRfaH fold from 40% to 22%. This is likely due to filtering out distant NusG-like proteins in the InterPro database (mean sequence identity to *E. coli* RfaH = 49.4%) that might be still present in ColabFoldDB, similar to previous works where NusG-like sequences were filtered out based on sequence-based secondary structure predictions before coevolutionary analysis (Galaz-Davison et al, 2022).

Finally, the predicted structures for the sequences from the Genomic Cluster exhibited the highest fraction of structures with an αRfaH fold (86%), with only 10% having a mixed α/β secondary structure content in their CTD, and merely 3% exhibiting a βRfaH fold (Figure 2C and Supplementary Figure 2C). This is a strong indicator that this set is indeed curated towards homologs very similar to *E. coli* RfaH, even though a small percentage of these orthologs are predicted by AF2 as monomorphic, most likely due to the higher sequence identity of these orthologs against *E. coli* RfaH (mean sequence identity to *E. coli* RfaH = 53.0%).

To determine if the predicted structures with an αRfaH fold from the different databases correspond to metamorphic representatives of this NusG subfamily, we performed two analyses. First, the structure of the isolated CTD of each protein was also predicted using ColabFold. With a handful exceptions, most of the isolated CTD sequences were predicted by AF2 to fold into the canonical βCTD structure (Figure 2D and Supplementary Figure 2), with 99% of them containing only β-strands.

**Figure 3:**
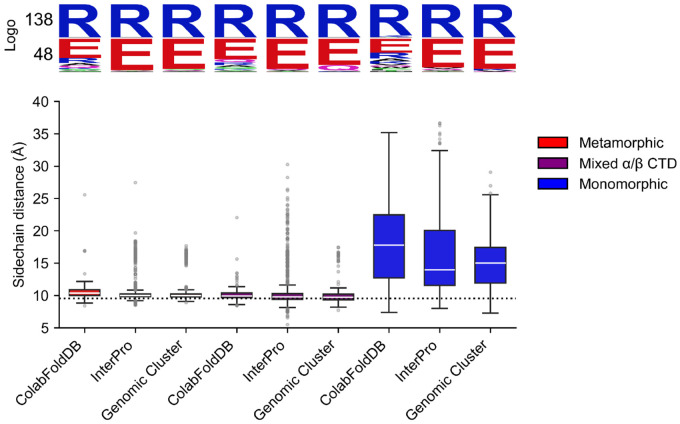
Cβ-Cβ sidechain distance between residues equivalent to the *E. coli* RfaH E48-R138 interdomain salt bridge in all predicted structures. For every structure prediction in each sequence set, residues in equivalent positions to E48 and R138 in *E. coli* RfaH, which are involved in a salt bridge that is critical for the stability of its autoinhibited state, were identified by pairwise sequence alignment to *E. coli* RfaH. Then, the Cβ-Cβ distance between these selected residues in each structure was determined. The box plots show the side chain distance distribution for each AF2-predicted dataset split by their classification (metamorphic in red, first 3 box plots on the left side; mixed α/β in purple, 3 box plots in the middle; monomorphic in blue, last 3 box plots on the right side). The sequence conservation logo of the residues involved in these interactions are displayed on top. The median is shown as a horizontal white line in each box plot, the lower and upper ends indicate the 25% and 75% interquartile range, and the whiskers extend to the percentile 1 and 99, with points representing outliers. The dotted line represents the Cβ-Cβ distance for the E48-R138 salt bridge in the best ranked AF2 structure of *E. coli α*RfaH.

Secondly, we determined the Cβ-Cβ sidechain distance in all predicted structures of full-length RfaH between residues equivalent to the interdomain E48-R138 salt bridge present in *E. coli* RfaH (see Methods). This salt bridge is known to stabilize the autoinhibited state, and its disruption via the E48A mutation leads to a 1:1 equilibrium between the autoinhibited and active state (Burmann et al, 2012). This analysis confirmed that the median and 25% lower interquartile distances for the predicted structures with an αRfaH fold from all databases were similar or slightly above the Cβ-Cβ sidechain distance between E48 and R138 (9.56 Å) in the best ranked predicted structure of *E. coli* αRfaH using ColabFold (Figure 3).

For the predicted structures in the αRfaH fold from ColabFoldDB, InterPro and Genomic Cluster, the median distances correspond to 10.34, 10.01 and 9.97 Å, respectively, and the distances in the 25% lower interquartile range are 10.00, 9.80 and 9.75 Å, respectively. For the structures predicted with a mixed α/β CTD from ColabFoldDB, InterPro and Genomic Cluster, the median distances were 9.93, 9.78 and 9.70 Å, respectively, and the distances in the 25% lower interquartile range are 9.64, 9.41 and 9.32 Å, respectively, which are even shorter than the best ranked predicted structure of *E. coli* αRfaH using ColabFold.

In contrast, the AF2-predicted structures of full-length RfaH homologs with a βCTD fold have median distances of 17.76 Å for ColabFoldDB, 13.96 Å for InterPro and 15.00 Å for Genomic Cluster, and the distances in the 25% lower interquartile range are 12.71, 11.55 and 11.91 Å, respectively. Notably, these differences are observed in these predicted structures regardless of the high conservation of the glutamic acid and arginine residues across all sequences in positions equivalent to *E. coli* RfaH residues 48 and 138, respectively (Figure 3).

Importantly, these results are well aligned with the differences in sequence identity for the predicted structures of RfaH homologs in their different structural classifications. Upon retrieving the best ranked predicted protein structures using the average per-residue local distance difference test (pLDDT, a confidence metric from AF2) (Jumper et al, 2021) of their CTDs as a proxy, we observed that the predictions of the αCTD and the βCTD folds exhibit higher pLDDT than the predictions of the CTD structures with mixed α/β secondary structure (Supplementary Figure 4).

Specifically, the median pLDDT for the αRfaH and βRfaH structures are 63.12 and 68.28 for ColabFoldDB, 62.29 and 62.39 for InterPro, and 61.16 and 56.16 for Genomic Cluster, respectively. Moreover, the upper 75% interquartile range has values of 67.26 and 80.85 for ColabFoldDB, 63.56 and 77.39 for InterPro, and 62.97 and 60.47 for Genomic Cluster, respectively. In contrast, the predictions of the mixed α/β have lower median and 75% interquartile range values of 48.84 and 56.66 for ColabFoldDB, 47.21 and 52.79 for InterPro, and 44.61 and 48.85 for Genomic Cluster, respectively (Supplementary Figure 4).

When analyzing the sequence identity percentage per structural classification against *E. coli* RfaH, the best αRfaH predicted structures from the ColabFoldDB, InterPro and Genomic Cluster databases have a median of 31.5, 47.5 and 48.7% sequence identity to *E. coli* RfaH, respectively. Conversely, the βRfaH predicted structures have a median of 29.6% for ColabFoldDB, 34.6% for InterPro and 38.9% for Genomic Cluster. Moreover, the 75% upper interquartile range has higher value for the αRfaH predicted structures from InterPro and Genomic Cluster than those from the βRfaH predicted structures (Supplementary Figure 3).

Altogether, these results strongly suggest that the predicted structures from all databases in the αRfaH fold represent the autoinhibited state unique to metamorphic RfaH homologs, whereas those predicted in the βRfaH fold represent putative extant monomorphic RfaH homologs.

### A mixed α/β CTD topology is observed in fold-switching simulations of *E. coli* RfaH

An interesting finding from the large-scale protein structure prediction of RfaH homologs from ColabFoldDB, InterPro and the Genomic Cluster is the significant number of predicted structures with mixed α/β secondary structure content in their CTD (Figure 2 and Supplementary Figure 2) in close proximity to the NTD, as ascertained by the sidechain distance between residues equivalent to the E48-R138 salt bridge in *E. coli* αRfaH (Figure 3). Moreover, predictions of the isolated CTD for RfaH homologs from all three databases mostly comprise β-strands (Supplementary Figure 2). These results, and our previous work using ColabFold to predict the structures of alanine scanning mutants of interdomain CTD residues in full-length *E. coli* RfaH, which also identified structures with such mixed α/β content in their CTDs (Tabilo-Agurto et al, 2025), strongly suggest that the mixed α/β topology originates from coevolutionary interactions retrieved from the MSA between NTD and CTD residues, rather than from coevolutionary signals within the CTD alone.

We sought to determine whether the mixed α/β CTD structures predicted by AF2 are also observed during the fold-switch of *E. coli* RfaH. To pursue this, we performed TMD (Schlitter et al, 1994), an out-of-equilibrium simulation technique to explore rare conformational transitions, such as the fold-switch of *E. coli* RfaH CTD in the context of the full-length protein, using an external potential over a collective variable that accurately describes this change in structure (see Methods). Then, we extracted all the structural ensembles explored during these TMD trajectories for analysis using AF2Rank (Roney & Ovchinnikov, 2022), a framework that repurposes AF2 as a scoring function to evaluate and rank the quality of protein structures.

**Figure 4:**
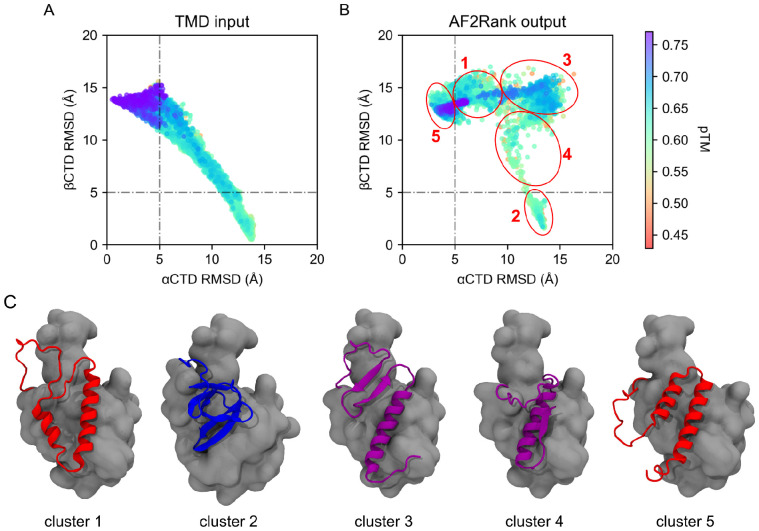
The structural diversity of RfaH is mirrored by fold-switching simulations of *E. coli* RfaH. (**A**) 2D projection of the RMSD and pTM calculated by AF2Rank for the input decoy structures obtained from nearly 200 TMD trajectories starting from either the autoinhibited or active state of RfaH. (**B**) 2D projection of the RMSD of the output structures refined by AF2Rank from the input TMD decoy structures, using the minimized structure of the autoinhibited and active states of *E. coli* RfaH. Each circle and number indicates the location around which a structural cluster was calculated. (**C**) Cartoon representation of the CTD structures for each cluster representative from the 5 relevant groups indicated in (**B**), with the NTD represented in surface representation in gray.

For this experiment, ensembles explored by 600 TMD trajectories of 100 ps from αRfaH to βRfaH and vice versa (300 in each direction) using implicit solvent (Supplementary Figure 5) were first clustered based on Principal Component Analysis (Supplementary Figure 6) to select the best 100 representative trajectories of the *E. coli* RfaH fold-switch in each direction (see Methods), and analyzed alongside the minimized structures of the *E. coli* αRfaH and βRfaH using AF2Rank after cycling once through the EvoFormer and Structure Module of AF2. These simulations were so short and were made using a range of forces so broad that only a small fraction of them reached the target state and are not amenable for thermodynamic analysis. However, their explored ensembles constitute a set of ~7,500 decoy structures from fold-switching simulations for calculating confidence metrics of the input structures, such as the predicted Template Modelling score (pTM) and mean pLDDT (Jumper et al, 2021), while also obtaining the minimized output structure after AF2 refinement based on its learned potential energy function (Roney & Ovchinnikov, 2022). This approach enables us to address how much these decoys resemble the mixed α/β CTD structures predicted by AF2 from our sequence databases and whether the learned energy function from AF2 converges to a subset of output structures from this set of initial decoys (Figure 4).

The AMBER-minimized autoinhibited and active states of *E. coli* RfaH were ranked favorably by AF2Rank, with pTM scores of up to 0.76 and 0.58, respectively, whereas the decoys obtained from the fold-switching simulations showed similar or lower pTM scores when compared to αRfaH or βRfaH, independent of the fold-switching TMD simulations being steered from the autoinhibited to the active state or vice versa (Figure 4A and Supplementary Figure 5).

After cycling each structure once through the AF2 network, the output structures were analyzed based on their structural similarity to either the αCTD or βCTD (Figure 4B). The distribution of these structures changes dramatically after being passed through AF2, with structural clustering analysis revealing 5 distinct groups: (i) cluster 1 (2,943 structures) corresponds to autoinhibited RfaH with an upside-down αCTD; (ii) cluster 2 (1,704 structures) - to active RfaH with the correctly folded βCTD; (iii) cluster 3 (1,380 structures) - to RfaH with the previously identified mixed α/β CTD fold in which β-strands β1-β2 are forming a β-sheet, whereas helix α2 is interacting with the NTD; (iv) cluster 4 (153 structures) - RfaH with a mixed α/β CTD fold in which only helix α2 and β-strand β-5 are folded, with helix α2 interacting with the NTD; and (v) cluster 5 (1,000 structures) - autoinhibited RfaH with the correctly oriented αCTD (Figure 4C).

We note that RfaH conformations with incorrectly oriented αCTDs have been already reported by us in 19 out of 100 simulations of *E. coli* RfaH using physical- and knowledge-based potentials going from the unfolded state to the autoinhibited αRfaH state (Galaz-Davison et al, 2021), whereas structures of *E. coli* RfaH with a mixed α/β CTD fold have been observed in AF2 prediction of alanine scanning mutants of interdomain CTD residues, some of which were shown to be constitutively (independently of *ops*) active (Tabilo-Agurto et al, 2025).

By tracing which input TMD decoys generated which AF2Rank output cluster (Supplementary Figure 7), it was possible to determine that the mixed α/β CTD in cluster 3 originates from input decoy structures that are dissimilar in terms of RMSD to both native CTDs and mostly obtained from TMD simulations of the fold-switch of RfaH from βCTD to αCTD, suggesting either disorder or partial β-folding. In contrast, the input decoys that are dissimilar to both native CTDs obtained from TMD simulations of the transition from αRfaH to βRfaH originates most of the structures with an upside down α-helical hairpin in cluster 1. Therefore, it can be concluded that TMD-derived structures deviating from αCTD and βCTD can be optimized by the structural module of AF2 into both of these CTD topologies.

When using an MSA instead, as in the case of the structure predictions of the RfaH orthologs from the different databases, a prediction of a mixed α/β CTD implies that the AF2 prediction is retrieving some signals related to the interdomain interactions with the NTD, making the distograms generated by the EvoFormer more diffuse (Supplementary Figure 8), thus causing the structural module to minimize RfaH into the mixed α/β CTD conformation.

### *In vivo* transcription-translation assays of RfaH homologs matches AF2 predictions

*E. coli* RfaH controls gene expression through contacts to RNAP via its NTD and the ribosome via its βCTD. Despite sharing only 21% sequence identity, RfaH and NusG make similar contacts to both macromolecular complexes (Kang et al, 2018; Wang et al, 2020b; Molodtsov et al, 2024) and RfaH orthologs from diverse bacteria, including the distant RfaH homolog from *V. cholerae* (43% sequence identity to *E. coli* RfaH), act similarly to *E. coli* RfaH in a heterologous *E. coli* system *in vitro* and complement *rfaH* deletion *in vivo* (Carter et al, 2004).

Thus, we selected candidates with either metamorphic, mixed α/β or monomorphic CTD from the Genomic Cluster of RfaH homologs (Supplementary Table 1), which were curated using HMMs of sequence conservation and Markov clustering of genome sequences (Wang et al, 2020a), to determine the functional phenotype of these structurally diverse RfaH orthologs using *in vivo* assays. We selected RfaH homologs exclusively from this curated dataset because these proteins were identified based both on their sequence conservation and on their presence in highly diverse gene neighborhood contexts (Wang et al, 2020a). Thus, the RfaH orthologs from the Genomic Cluster are more likely to retain canonical RfaH-like regulatory functions, such as transcription-translation coupling, as compared to those identified solely by sequence conservation (e.g., from ColabFoldDB or InterPro) despite their divergent sequences.

For these experiments, we used a well-established *in vivo* luciferase reporter assay (Supplementary Table 2), in which one plasmid encodes the *Photorhabdus luminescens luxCDABE* operon positioned downstream of the *P_BAD_* promoter, while a second compatible plasmid expresses RfaH (or its variant) from an IPTG-inducible *P_trc_* promoter (Belogurov et al, 2010), and both plasmids are co-transformed into an *E. coli* strain lacking chromosomally encoded RfaH (DH5α Δ*rfaH*). The *in vivo* luminescence activity levels are normalized by cell growth, ascertained by optical density at 600 nm (OD_600_, Supplementary Figure 9).

**Figure 5:**
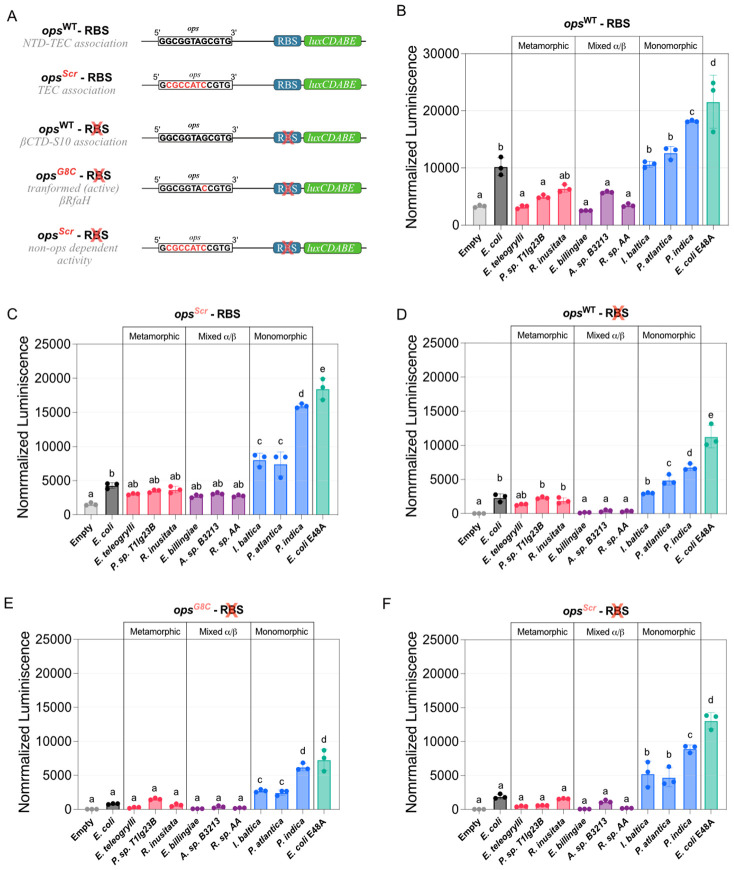
Cellular assays of RfaH homologs’ effects on gene expression. (**A**) Reporter constructs used for exploring different aspects of RfaH action on transcription-translation coupling in *E. coli* DH5α Δ*rfaH* cells. The *in vivo* activity is determined by the levels of luminescence generated by the *luxCDABE* operon encoded on each plasmid in response to RfaH-regulated expression and the features of the *ops* and the presence/absence of RBS upstream of the *luxC* gene: wild-type *ops* (*ops*^WT^) and RBS (**B**), scrambled *ops* (*ops*^Scr^) and RBS (**C**), *ops*^WT^ and absent RBS (**D**), G8C mutant of *ops* (*ops*^G8C^) and absent RBS (**E**), and *ops*^Scr^ and absent RBS (**F**). Three controls per group were used in these assays: P*trc* plasmids encoding WT *E. coli* RfaH, its constitutively active E48A mutant, and an empty vector. Each bar represents the average raw luminescence level of six technical replicates of three colonies, normalised by cell density. The error bar shows the standard deviation. A one-way Analysis of Variance (ANOVA) was used to assess statistical significance, with all samples in an assay with a given luminescence construct compared to each other. RfaH homologs sharing the same letter are not significantly different (p > 0.05), whereas groups with different letters are significantly different (p < 0.05).

The regions upstream of the *lux*CDABE operon can be designed to test different features of RfaH activity *in vivo* (Figure 5A). For example, *lux* expression depends on RfaH when the RBS is absent, requiring RfaH-mediated ribosome recruitment when the CTD reaches the β-folded state (Burmann et al, 2012). Expression also relies on the *ops* element when metamorphic RfaH is used because fold-switching activation of *E. coli* RfaH is triggered by interactions of RfaH with *ops*-paused RNAP (Burmann et al, 2012). The absence of both a functional *ops* and an RBS leads to very low levels of *lux* expression in the presence of *E. coli* RfaH, comparable to an empty vector lacking RfaH (Figure 5E and 5F), as RfaH fails to load and fold-switch, opening a path to NusG/Rho-mediated transcriptional termination (Cardinale et al, 2008; Lawson et al, 2018).

Conversely, RfaH variants with destabilized NTD-αCTD interactions, such as E48A and L142A, exhibit higher activity without depending on the *ops* sequence (Burmann et al, 2012; Tabilo-Agurto et al, 2025), as demonstrated by the use of *ops* mutants that induce RNAP pausing but fail to recruit RfaH, such as a scrambled *ops* sequence (*ops*^Scr^) (Belogurov et al, 2007) and the *ops* G8C mutant (*ops*^G8C^) (Svetlov et al, 2018). While autoinhibited RfaH can only be loaded at the *ops* WT site (*ops*^WT^), constitutively active variants have multiple opportunities to engage RNAP throughout transcription (Burmann et al, 2012; Tabilo-Agurto et al, 2025).

The assay results suggest that putative metamorphic orthologs exhibit *E. coli* RfaH-like activity primarily in the genetic architecture with *ops*^WT^ lacking RBS (Figure 5D). Among these, only *Erwinia billingiae* RfaH displayed intermediate activity levels between the empty vector and *E. coli* RfaH. Notably, no activity was observed with any non-canonical *ops* sequences (Figures 5E and 5F), further supporting the requirement of the *ops* signal to trigger domain dissociation and the transition to the active βCTD state. However, the efficiency of these orthologs in this heterologous system appears limited: under leaky, low-level expression, statistical analysis showed no significant difference compared to the control, even when all regulatory elements are present in the *ops*^WT^ context with RBS (Figure 5B). Interestingly, the activity of these RfaH orthologs is restored when their expression is increased due to presence of IPTG to induce protein overexpression under the same genetic context of *ops*^WT^ with RBS (Supplementary Figure 10). These results suggest that their low activity under more stringent conditions is attributable to suboptimal recruitment or impaired fold-switching due to lower binding affinity to RNAP in the heterologous *E. coli* system.

Intriguingly, in cases where the sequence is classified as monomorphic, and the protein is consequently expected to be constitutively active, the orthologs exhibited activity across all tested *ops* contexts, including conditions lacking an RBS (Figure 5B-F). Moreover, their activity levels were comparable to the *E. coli* RfaH E48A mutant, which disrupts a key salt bridge at the NTD:CTD interface, resulting in a significant active population *in vivo* without requiring activation by *ops* signal. When comparing their sequences against *E. coli* RfaH, all monomorphic orthologs were found to lack a few key *ops*-binding residues (Supplementary Figure 11). Furthermore, these proteins have diverged evolutionarily from NusG, as they lack the conserved sequences in loop 2 required for Rho binding (Supplementary Figure 11), and they can regulate the expression of this long operon even when the RBS is absent. Consequently, this study provides the first indirect evidence of putative, constitutively active RfaH proteins present in other bacteria whose structure predictions indicate that they will be monomorphic and folded in the canonical NusG-like structure.

Finally, the mixed α/β topology likely reflects fold instability, resulting in lower activity than *E. coli* RfaH in RfaH:S10-dependent translation. Indeed, this group of orthologs showed reduced activity, particularly upon removal of the RBS (Figures 5D-F), a condition where stable βCTD folding is essential for functional coupling. However, this observed lack of activity may also be attributed to expression or solubility issues, as even under conditions that induce protein expression, their activity recovery in the *ops*^WT^ with RBS architecture was less pronounced than that of their metamorphic counterparts (Supplementary Figure 10).

### AF2 classification relates to phylogeny and genome location of RfaH-dependent operons.

Observed *in vivo* differences in RfaH activity matched their predicted CTD folding, prompting us to explore the phylogeny of these sequences. Since AF2 uses coevolutionary information from the MSA, which is generated by pairing homologous sequences close to the input query, some evolutionary information is already incorporated into structure prediction. This is because the MSA guides the predictions via the Evoformer; therefore, alignments including different homologs would result in sampling different parts of the conformational space for the query sequence, as reported in previous works (Wayment-Steele et al, 2024; Lee et al, 2025).

**Figure 6:**
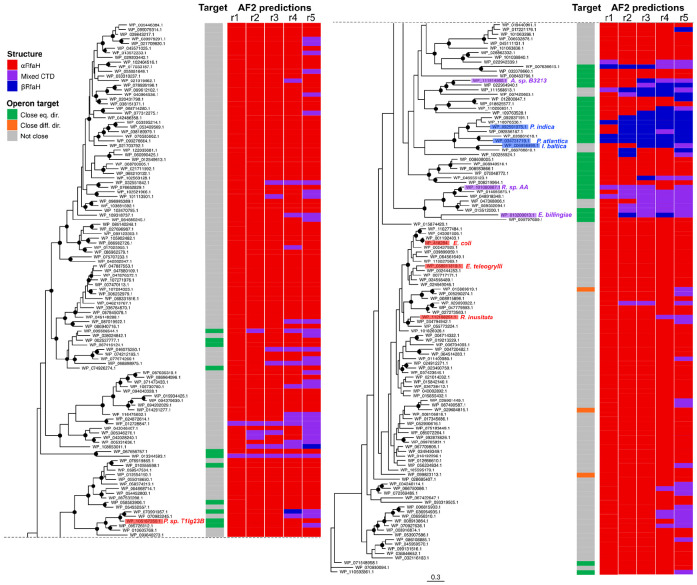
AF2 predictions are grouped by phylogeny and match long operon vicinity. RfaH-like sequences within the Genomic Cluster were used to infer a phylogenetic tree, using *E. coli* NusG as the outgroup. Nodes with Shimodaira–Hasegawa-like approximate likelihood ratio test (SH-aLRT) ≥ 80% and ultrafast bootstrap (UFBoot) ≥ 95% support are highlighted with black circles. The potential target operon of each RfaH ortholog was identified and classified as either distant (grey) or near to the *rfaH* genome-encoded gene, and if so, whether it was in the same (green) or different (orange) direction (see Methods). The 5 models obtained for each RfaH ortholog used to construct the tree from the AF2 predictions are ranked from best (r1) to worst (r5) and color coded as metamorphic (red), mixed α/β CTD (purple) or monomorphic (blue).

As expected from the large-scale structure predictions herein and the experimental validations using a subset of RfaH orthologs, we generated a maximum likelihood phylogenetic tree using a MSA of the proteins from the Genomic Cluster using IQ-TREE 2 (Minh et al, 2020), which shows that similar structural predictions are shared among related members of distinct clades, particularly resulting in large clades for most of the metamorphic predictions, and smaller clades containing the mixed α/β and monomorphic members (Figure 6).

The uncontrolled *ops*-independent recruitment of monomorphic RfaH could pose a challenge for the survival of the host bacterium because RfaH binds to RNAP with higher affinity than NusG (Kang et al, 2018) and inhibits Rho-mediated termination (Sevostyanova et al, 2011), which is an essential function of *E. coli* NusG (Cardinale et al, 2008; Lawson et al, 2018). A previously proposed ancestral recruitment mechanism, wherein the RfaH NTD is recruited to the transcribing RNAP while the CTD is being made by the ribosome (Belogurov et al, 2009), could explain how RfaH could be “selectively” targeted to avoid the lethal NusG exclusion. Since this mechanism requires that RfaH acts *in cis*, we investigated operons located in the vicinity of the *rfaH* gene within the genomes of these organisms (see Methods).

By tracking the relative location of *rfaH* with respect to neighbouring 30 genes and their operon organization, it can be seen that the operons surrounding genes of most metamorphic members are not adjacent to the *rfaH* gene and thus are likely not regulated by RfaH (Figure 6). This pattern is expected because *E. coli*-like metamorphic RfaH proteins have evolved to act on their targets *in trans*, by becoming activated only upon binding to their cognate (*ops*-like) target (Zuber et al, 2018). In that case, autoinhibition ensures that RfaH is able to control far-away operons but does not interfere with the constitutive and essential function of NusG function.

A different pattern is observed in several of the species with predicted RfaH structures with mixed α/β CTDs and in most of the monomorphic RfaH clade organisms with canonical NusG-like states, for which a long operon is located in the immediate genomic neighborhood or contains an *rfaH* gene (Supplementary Figure 12). These neighbor operons in monomorphic proteins are indeed mostly populated with virulence factors such as LPS biosynthesis machinery (Table 1) and secretion system proteins, which are well known targets for RfaH (Bailey et al, 1997).

## DISCUSSION

Metamorphic proteins present a challenge for DL structure prediction algorithms as the predominant fold is commonly observed. AF2 in its default implementation is not exempt from this issue (Chakravarty & Porter, 2022), prompting the use of different approaches to discover alternate structures, such as sequence-based (Wayment-Steele et al, 2024) and energy-based MSA clustering (Guan et al, 2024) or random sampling approaches (Lee et al, 2025). An alternative approach, as utilized herein, is to cluster the sequences from a given protein family, predict their three-dimensional structures, and analyze their structural differences. If the MSA is representative of the orthologs in the family, so that each clade member can be predicted accurately, then the structural differences therein are likely arising from changes in sequence conservation and coevolution.

In this work, we used RfaH, a metamorphic ubiquitous transcription factor for which a wealth of functional (Belogurov et al, 2010; Burmann et al, 2012; Shi et al, 2017; Tabilo-Agurto et al, 2025), structural (Burmann et al, 2012; Zuber et al, 2018; Kang et al, 2018; Zuber et al, 2019, 2024), and *in silico* modeling (Wayment-Steele et al, 2024; Tabilo-Agurto et al, 2025; Lee et al, 2025) and simulation data (Ramírez-Sarmiento et al, 2015; Gc et al, 2015; Seifi et al, 2021; Galaz-Davison et al, 2021; Appadurai et al, 2021; Zuber et al, 2024; González-Higueras et al, 2024) are available to evaluate the potential of this approach. Our present results argue that the prediction of monomorphic and mixed secondary structures of fold-switching CTD in the RfaH family does not necessarily represent failures in AF2 predictions. Instead, we posit that these “unexpected” folds correspond to RfaH orthologs that lack a fold-switching mechanism of fully-evolved RfaH and instead assume the constitutive active state. Our *in vivo* activity assays, phylogenetic analysis, and examination of genomic neighborhoods support the existence of previously hypothesized monomorphic RfaH orthologs which could be recruited to their targets in *cis*.

**Figure 7:**
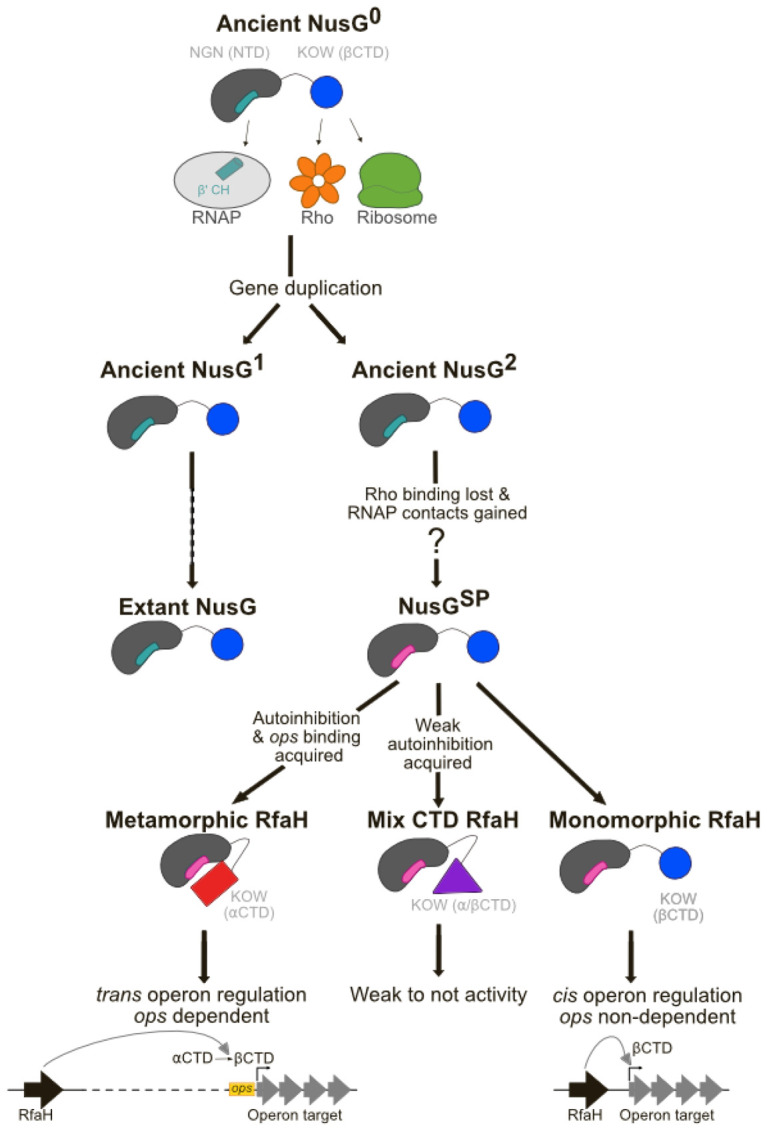
Step-wise structural and functional path from ancient to extant RfaH orthologs.

To the best of our knowledge, this is the first report to describe putative monomorphic RfaH orthologs with distinct functional patterns, diverging from the known metamorphic RfaH. Based on our findings, we propose a new evolutionary branch within the RfaH family, in which some orthologs have bypassed the metamorphic requirement to achieve constitutive activity, likely as an adaptation to specific genomic and regulatory contexts (Figure 7).

Previous reports suggested that RfaH family sequences with non-metamorphic features were likely from its non-switching paralog NusG (Galaz-Davison et al, 2022; Porter et al, 2022). RfaH and NusG possess a mixture of parallel (transcription processivity and coupling to translation) and orthogonal (Rho-dependent transcription termination) functions (Wang & Artsimovitch, 2020), and sequence alone is insufficient to discern the homology relationship within this family. Here, we focused on RfaH homologs that were clustered using HMMs alongside *E. coli* RfaH and expected to be metamorphic (Belogurov et al, 2009; Wang et al, 2020a). However, our results suggest that some members of this group are monomorphic, and their genomic context is different from those of both the canonical metamorphic RfaH and the monomorphic NusG. We find that, like RfaH, its monomorphic homologs activate expression of a horizontally-acquired *P. luminescens lux* operon. This activity contrasts with the canonical function of NusG, which aids Rho in termination transcription of foreign DNA (Lawson et al, 2018). Thus, the putative monomorphic RfaH homologs act as RfaH and not as NusG.

RfaH promotes gene expression by counteracting Rho/NusG silencing through many mechanisms, all of which require RfaH activation through the *ops*-mediated fold switch (Wang & Artsimovitch, 2020). The requirement for *ops* can be bypassed by weakening the NTD-CTD interactions to stabilize the active (monomorphic) state, as observed with the constitutively active (*ops*-independent) E48A RfaH variant (Burmann et al, 2012). We show that the putative monomorphic RfaH homologs activate *lux* expression similarly to E48A (Figure 5) and are encoded near their putative target operons (Figure 6), supporting the idea that these proteins are not autoinhibited and achieve selective recruitment *in cis*.

Another predicted state with mixed α/β CTD structure was observed as part of the AF2 predictions of RfaH homologs. Our analysis of the transition between αRfaH and βRfaH for *E. coli* RfaH through TMD simulations, which were then input to AF2Rank, revealed the presence of five well-defined structural clusters, two of which correspond to its autoinhibited and active states, and three clusters representing alternative conformations: (i) RfaH with an upside-down αCTD, RfaH with only helix α2 and β-strand β5 folded and, importantly, the mixed α/β state observed in 9% of the predicted structures of RfaH homologs. Before passing through AF2Rank, the structures exploring the transition between native states display a high RMSD to both experimental structures, likely exploring high energy parts of the RfaH free energy landscape, as shown previously (Galaz-Davison et al, 2021).

Coincidentally, after passing through the AF2 architecture, the RfaH structures with mixed α/β CTD remain at the high energy part of the landscape and display lower pTM and lower mean pLDDT than proteins with αRfaH and βRfaH folds. Although these confidence metrics are not directly related with experimental stability, together with low *in vivo* activity of the mixed α/β CTD variants without RBS (Figure 5), they are consistent with an unstable fold. From an evolutionary standpoint, the instability caused by the presence of the NTD can be interpreted as a signal of structural ambiguity in protein sequences relating to emerging folds and fold-switching (Tian & Best, 2020), suggesting that mixed α/β orthologs are still undergoing structural and functional diversification towards *E. coli*-like, fully evolved RfaH.

Phylogenetic analysis reveals that AF2 predictions with similar fold propensities are clustered in well-defined clades. Although AF2 and phylogenetic inference are separate experiments, a correlation between them is expected for structural predictions that are based on evolutionary information. This correlation implies that the sequence variability was sufficient to detect the mixed α/β and monomorphic RfaH homologs in the AF2 protein structure predictions, which have different genomic contexts and *in vivo* activity profiles, supporting the idea that their structural diversity is associated with distinct functional outcomes.

## METHODS

### Large-scale protein structure prediction

Amino acid sequences for *E. coli* RfaH homologs were retrieved from three databases: (a) ColabFoldDB (Mirdita et al, 2022) (468 sequences), accessed on January 16 2026 via MMseqs2 (Steinegger & Söding, 2017) using the sequence of *E. coli* RfaH (UniProt accession ID P0AFW0) as input for ColabFold v1.5.5 (Mirdita et al, 2022) and filtered based on a minimum sequence coverage of 95%, a bit-score of 140 and a maximum e-value of 1e-30; (b) InterPro (Blum et al, 2025) (family entry IPR010215, 3,058 sequences), accessed on January 3 2024; and (c) a previously reported list of 416 RfaH sequences (Cluster 1) curated by HMMs with known genomic context that were classified by Markov clustering and include *E. coli* RfaH (Genomic Cluster, hereafter) (Wang et al, 2020a). In the case of the ColabFoldDB sequences, these were filtered to eliminate duplicate sequences between this database and InterPro using CD-HIT v4.8.1 (Fu et al, 2012), and *E. coli* RfaH (UniProt accession ID P0AFW0) was manually added.

These 3,942 RfaH-like sequences were used as input for DL-guided protein structure prediction using a local version of ColabFold v1.5.5 (Mirdita et al, 2022) (LocalColabFold, available at https://github.com/YoshitakaMo/localcolabfold) using default parameters: 1 seed, 5 models, alphafold2_ptm model for monomers, 3 recycles, no templates, MSAs generated using MMseqs2 (Steinegger & Söding, 2017). The resulting 19,710 structures were analyzed using TM-align v20220412 (Zhang & Skolnick, 2005) to calculate the TM-score (Zhang & Skolnick, 2004), a metric to assess the similarity between two protein structures ranging from 0 (no match) to 1 (exact match), and the root mean square deviation (RMSD) in Å for the full-length protein and their CTDs, using the best ranked predicted structure of *E. coli* αRfaH and its αCTD and the experimental structure of the βCTD of *E. coli* (PDB ID 2LCL) as references. The per-residue secondary structure of the CTD of the predicted structures was analyzed using STRIDE v1.6.4 (Frishman & Argos, 1995). ColabFold was also used to obtain the distograms (set at a 1 nm cutoff) of *E. coli* RfaH in the active and autoinhibited states, obtained using 0 and 3 recycles, respectively, as well as the distograms of the 9 selected RfaH orthologs from the Genomic Cluster for experimental characterization after 3 recycles.

Frequency histograms of the percentage of α-helices (classified as ‘H’ in STRIDE) and β-strand (classified as ‘E’ in STRIDE) for all predicted structures from all databases were first used to determine potential cutoffs to distinguish between metamorphic αRfaH, monomorphic βRfaH and RfaH with mixed α/β CTD (Supplementary Figure 1). Based on this analysis and the histograms in the plots of α-helical and β-strand secondary structure, metamorphic αRfaH were classified based on having a percentage of α-helices > 32.5% and < 2.5% of β-strands, monomorphic βRfaH on having a percentage of β-strands > 30.0% and < 2.5% of α-helices, and RfaH with mixed secondary structure in their CTD on having a percentage of α-helices and β-strands > 2.5%. Other secondary structure ranges were classified as ‘none’. The data was further manually curated based on visualization of the predicted structures, which are presented throughout the manuscript through images generated using VMD v1.9.3 (Humphrey et al, 1996).

Additionally, salt-bridge distances were calculated between residues equivalent to the E48-R138 pair in E. coli RfaH for all predicted structures by determining these residues from each protein based on a MSA generated for each database, including E. coli RfaH, using MAFFT v7.526 (Katoh & Standley, 2013) in auto mode, and a custom python script to get the coordinates of the Cβ atoms of the sidechain of these residues (Cα in the case of glycine) and measure their distance. Lastly, sequence logos for the residues in these positions of the alignment with respect to E. coli RfaH were calculated from the MAFFT-generated MSAs for each database using Logomaker v0.8 (Tareen & Kinney, 2020).

### TMD simulations

The full-length structures of the autoinhibited and active states of *E. coli* RfaH were obtained from a previous work (González-Higueras et al, 2024). Briefly, αRfaH was generated using the experimentally solved structures of its autoinhibited state (PDB 5OND, chain B, NTD; PDB 2OUG, chain B, αCTD), whereas for βRfaH, the αCTD was replaced by the experimentally solved structure of βCTD (PDB 2LCL). Missing linker residues between the NTD and CTD were added using MODELLER v9.23 (Webb & Sali, 2016). Both models were energy minimized after being solvated in a cubic box of 1.0 nm of padding with TIP3P water molecules and neutralized with counterions using GROMACS v4.5.3 (Pronk et al, 2013) and the Amber ff99SB-ILDN force field (Lindorff-Larsen et al, 2010) using the steepest descent method.

The TMD simulations presented herein were performed similarly to a previous work from our group (Ramírez-Sarmiento et al, 2015), in which the collective variable that steers RfaH *E. coli* from one native state into another is defined by applying an external potential to minimize the root mean square deviation (RMSD) of the protein coordinates relative to the target final structure during the simulation trajectory, according to equation 1:

(eq. 1) $$E_{TMD}=\frac{1}{2}\cdot k\cdot N\cdot {\left(\text{RMSD}(t)-{\text{RMSD}}_{\text{target}}\right)}^{2}$$

Where k is the force constant, N is the number of atoms used as a collective variable during the TMD simulation, ${\text{RMSD}}_{\text{target}}$ is the target RMSD and RMSD(t) is the calculated frame at each timestep during the simulation.

The main differences with our previous work are: (i) the use of the RMSD for the backbone atoms of residues 110-162 in full-length *E. coli* RfaH as collective variable (totaling 252 atoms); and (ii) the use of an implicit solvent model, which reduces the computational cost and, consequently, allows to perform multiple TMD simulations using different external potential force constants to steer *E. coli* RfaH towards either the autoinhibited or the active state.

Specifically, we utilized tleap from AmberTools v22.3 (Case et al, 2023) to parameterize our simulations systems for *E. coli* αRfaH and βRfaH using the ff19SB force field (Tian et al, 2020) and the GB-Neck2 implicit solvent model with mbondi3 intrinsic radii to accurately represent the solvation environment (Nguyen et al, 2013) to perform TMD simulations using AMBER 22 (Case et al, 2025). First, we minimized each system for 2,500 steps using a steepest descent gradient, followed by 47,500 steps of conjugate gradient, before performing 300 short (100 ps) TMD simulations from the autoinhibited to the active state and vice versa (600 TMD simulations total) at a temperature of 298 K controlled by a Langevin thermostat and the SHAKE algorithm to constrain hydrogen-atom bond lengths to allow simulations with a time step of 2 fs, using randomly selected force constants ranging from 1.0×10^−4^ to 3×10^−2^ kcal/(mol·Å^2^) applied over the backbone atoms of residues 110-162, while aligning the protein during the simulation based on the backbone atoms of the NTD, with a target RMSD of 0 Å to the final reference structure. Frames were collected every 2 ps, thus generating 50 frames per trajectory for analysis.

To select a representative number of TMD trajectories that explored all possible configurations across the fold-switch from αRfaH to βRfaH and vice versa, a universal Principal Component Analysis (PCA) map was constructed using the 10 trajectories from each set of TMD simulations in each direction of the RfaH fold-switch that reached the minimum RMSD using cpptraj v6.4.4 from AmberTools v22.3 (Case et al, 2023). With this data, a coordinate covariance matrix was calculated using the Cα atoms of the fold-switching CTD (residues 110-162), while the NTD was used as a rigid-body reference for alignment to remove translational and rotational motions. The covariance matrix was diagonalized to obtain the eigenvectors, upon which all 600 trajectories were projected onto the first two principal components (PC1 and PC2) of this universal PCA map (Supplementary Figure 6), allowing for a direct structural comparison of the hysteresis between the different directions of the TMD simulations.

Lastly, to isolate the most representative and structurally diverse transition pathways, *k*-means clustering was performed on the projected PCA data. For each of the 300 TMD simulation datasets, the trajectory frames were clustered into *k* = 100 distinct groups based on their Euclidean distance in the PC1/PC2 subspace. The centroid of each cluster was identified, and the specific trajectory containing the centroid frame was selected as a representative pathway. This process yielded a curated dataset of 100 TMD trajectories for the simulations from αRfaH to βRfaH (3,750 decoy structures) and 99 TMD trajectories for the simulations from βRfaH to αRfaH (3,700 decoy structures), ensuring broad sampling of the fold-switching transitions in each direction while removing redundant paths.

### Decoy analysis using AF2Rank

The original Jupyter notebook of AF2Rank for Google Colab (Roney & Ovchinnikov, 2022), available on GitHub (https://github.com/jproney/AF2Rank), was modified to analyze all 7,450 decoy structures generated by TMD using both the AMBER-minimized αRfaH and βRfaH states of *E. coli* RfaH as native states. AF2Rank was run using default parameters (1 recycle, alphafold_ptm model and 1 model per input decoy) as well as masking the sequence and sidechains of the structures, in order to prevent AF2 from using its knowledge of evolutionary couplings or specific amino acid sequences to weigh in when evaluating the physical plausibility of the decoy structures and generating the output structures after passing through the EvoFormer and Structure Module.

After obtaining the output structures from AF2Rank, they were subjected to *k*-means clustering based on the Cα RMSD of their CTDs (residues 110–162) against the CTDs of the AMBER-minimized αRfaH and βRfaH states of *E. coli* RfaH. Structures were grouped in five clusters (*k* = 5) and the medoid structure from each cluster was selected as representative structure for visualization using VMD v1.9.3 (Humphrey et al, 1996).

### *In vivo* luminescence assay

DH5α Δ*rfaH* (IA149) was co-transformed with two plasmids. One chloramphenicol-resistant plasmid encoded one of the 9 selected RfaH orthologs from the Genomic Cluster (Supplementary Table 1) with at least 2/5 AF2 models predicted in the metamorphic αRfaH state (GenBank accession ID WP_058911819.1, *Erwinia teleogrylli*; WP_105167350.1, *Pseudoalteromonas sp.* T1lg23B; WP_112168251.1, *Rahnella inusitata*), the monomorphic βRfaH state (WP_006956899.1, *Idiomarina baltica*; WP_034731719.1, *Pseudidiomarina atlantica*; WP_092591975.1, *Pseudidiomarina indica*) or with a mixed α/β CTD (WP_013200013.1, *Erwinia billingiae*; WP_111914955.1, *Aliidiomarina sp.* B3213; WP_101080081.1, *Rahnella sp.* AA), *E. coli* RfaH or constitutively active *E. coli* RfaH E48A downstream of a *P_trc_* promoter that can be used to induce protein overexpression by addition of Isopropyl-β-D-thiogalactopyranoside (IPTG). The second ampicillin-resistant plasmid encoded the *luxCDABE* operon downstream of several genetic constructs of *ops* (*ops*^WT^, *ops*^G8C^, *ops*^Scr^) with and without an RBS site under the control of a *P_BAD_* promoter (Supplementary Table 1).

Single colonies co-transformed with both plasmids were grown overnight (ON) at 37°C in liquid LB medium supplemented with antibiotics (30 μg/mL chloramphenicol and 100 μg/mL carbenicillin). The following day, saturated cultures were diluted to an initial optical density at 600 nm (OD_600_) of 0.05 in 3 mL of fresh LB medium supplemented with 0.1% glucose and the same concentration of antibiotics. Cells were grown until reaching a similar late exponential phase OD_600_ ≈ 0.7-0.8 (Supplementary Figure 9). For IPTG-induced conditions (Supplementary Figure 10), cultures were grown to an OD_600_ of 0.2–0.3 and then induced with 0.2 mM IPTG for 1 h. The final OD_600_ was measured using a UV/Vis spectrophotometer (Jasco V-730, Jasco Inc. Japan), and luminescence was recorded in black 96-well plates using a multi-mode plate reader (Synergy HTX, BioTek). Three biological replicates were performed per RfaH protein per condition, as well as for a negative control with a chloramphenicol-resistant plasmid not encoding any RfaH protein (empty).

### Phylogenetic tree inference

The sequences from the genomic cluster were subjected to clustering using CD-HIT v4.8.1 (Fu et al, 2012) at a sequence identity cutoff of 85%. An MSA of the clustered sequences, in which *E. coli* NusG was included, was generated with MUSCLE (Edgar, 2004), available at the EMBL-EBI Job Dispatcher web server (Madeira et al, 2024). After several iterations of manual curation, the final alignment, comprising 231 sequences, was used to infer the phylogenetic tree by maximum likelihood using IQ-TREE 2 (Minh et al, 2020) with the built-in automatic best-fit evolutionary model selection, which in this case was determined to correspond to LG (Le & Gascuel, 2008) with a proportion of invariant sites and gamma-distributed rate heterogeneity across sites (LG+I+G4). Maximum-likelihood tree inference was performed with an extended search of 2,000 trees, and branch support was assessed using 2,000 replicates of the Shimodaira–Hasegawa-like approximate likelihood ratio test (SH-aLRT) and 2,000 ultrafast bootstrap (UFBoot) replicates. Support values from both methods were mapped onto the resulting maximum-likelihood phylogeny. *E. coli* NusG was used as the outgroup to root the tree.

### Genomic context analysis

RfaH homologs from the Genomic Cluster were submitted to TREND (Gumerov & Zhulin, 2020) (https://trend.evobionet.com/) to extract neighboring genes (up to 15 genes on each side). The resulting datasets were downloaded and analyzed. Gene neighbors were sorted by genomic coordinates, and we applied the following criteria to determine whether an RfaH homolog is located near its potential target operon:

Step 1 – Identification of long operons. Since RfaH is required for transcription of long operons, a long operon was first defined based on the following conditions: (i) all constituent genes have the same transcriptional direction; (ii) the intergenic distance between adjacent genes is ≤200 bp; and (iii) the total length of the operon exceeds 5,000 bp.

Step 2 – Assessment of distance between RfaH homolog and operon. If an operon was identified near an RfaH homolog, we calculated the genomic distance to determine whether they are in close proximity. Three scenarios were considered: (i) (RfaH homolog and the operon has same "+" direction) or (RfaH homolog has "−" and the operon has "+" direction). To be close to the operon, absolute value of ((the end of RfaH homolog) - (start of first gene of operon)) < 1000 bp; (ii) (RfaH homolog and operon has same "−" direction) or (RfaH homolog has "+" and operon has "−" direction). Note that in the case of an operon showing “−“ orientation, the last gene of the operon is nearest to the promoter because the genes are ordered according to coordinates. The absolute value of ((the start of RfaH homolog) - (end of last gene of operon)) < 1000 bp. (iii) If the RfaH homolog is located inside the operon, it is automatically considered adjacent to it.

## Supplementary Material

Supplement 1

## Figures and Tables

**Table 1.** Lipopolysaccharide-related gene in operon next to the different RfaH orthologs

*Category*	Total	Next to operon	LPS in Operon
Metamorphic	212	17	15
Mixed α/β	12	8	1
Monomorphic	10	7	7

A summary of genomic contexts of RfaH orthologs classified according to the AF2 predictions. The numbers indicate how many *rfaH* genes are next to an operon and whether the operon contains lipopolysaccharide biosynthesis genes.

## Data Availability

Protein structure predictions of RfaH orthologs using ColabFold, TMD decoys for AF2Rank, Jupyter Notebooks for reproducing our data analysis using Google Colab and results from the analysis of all protein structures is available in Zenodo (10.5281/zenodo.19041374).
