# Supplementary material for: Exploration of the structural and functional diversity in the metamorphic RfaH subfamily
Source: bioRxiv. 2026 Mar 18:2026.03.16.712203. Preprint. [Version 1] doi: 10.64898/2026.03.16.712203 (PMC13015549; doi:10.64898/2026.03.16.712203)
Supplement: Supplement 1 [file media-1.pdf]

SUPPLEMENTARY INFORMATION

Exploration of the structural and functional diversity in the metamorphic RfaH subfamily

Cyndi Tabilo-Agurto[1,2†], Bastián González-Bustos[1,2†], Javiera Reyes[1,2], Bing Wang[3], Damaris Palomera[1,2], Verónica Del Río-Pinilla[1,2], Camila Neira-Mahuzier[1,2], Valentina Vera-Sandoval[1,2], Irina Artsimovitch[3], Pablo Galaz-Davison[4*], César A. Ramírez-Sarmiento[1,2*].

[1]Institute for Biological and Medical Engineering, Schools of Engineering, Medicine and Biological Sciences, Pontificia Universidad Católica de Chile, Santiago 7820436, Chile.
[2]ANID – Millennium Science Initiative Program, Millennium Institute for Integrative Biology (iBio), Santiago 833150, Chile.
[3]Department of Microbiology and Center for RNA Biology, The Ohio State University, Columbus, OH 43210, USA.
[4]Center for Bioinformatics, Simulation and Modeling, School of Engineering, Universidad de Talca, Talca 3460000, Chile.

[†]These authors contributed equally.

*Corresponding authors.

This Supplementary Information contains:

- 12 Supplementary Figures
- 2 Supplementary Tables
- 4 Supplementary References

SUPPLEMENTARY FIGURES

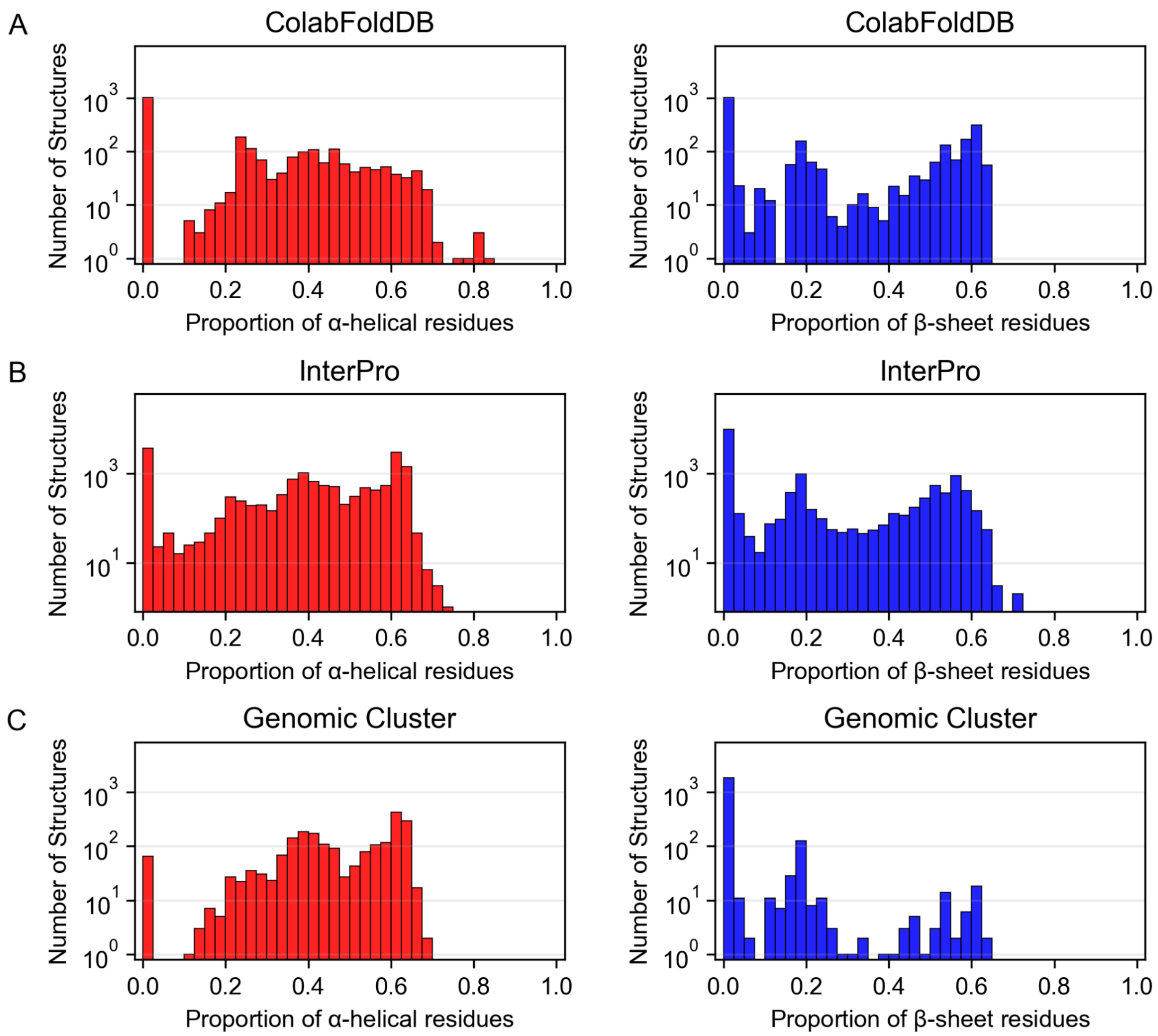

Supplementary Figure 1 Distribution of secondary structure in the CTD of structure predictions of full-length RfaH homologs. Distribution of α-helical (red) and β-strand (blue) secondary structure content analyzed using STRIDE for the 19,710 predicted structures of ColabFoldDB (**A**, 2,340 structures), InterPro (**B**, 15,290 structures), and Genomic Cluster (**C**, 2,080 structures), with the y axis corresponding to the number of structures with a given proportion of secondary structure in logarithmic scale. A small dip in the proportion of secondary structure is observed at 0.325 for α-helical residues and 0.300 for β-strands (more clearly observed for the plots in **C**). We used these fractions as thresholds to distinguish between αRfaH (fraction of α-helical structure > 0.325 and β-strand ~0), βRfaH (fraction of β-strand structure > 0.300 and α-helical ~0) or mixed α/β secondary structure (fraction of α-helical structure and β-strand structure > 0.025).

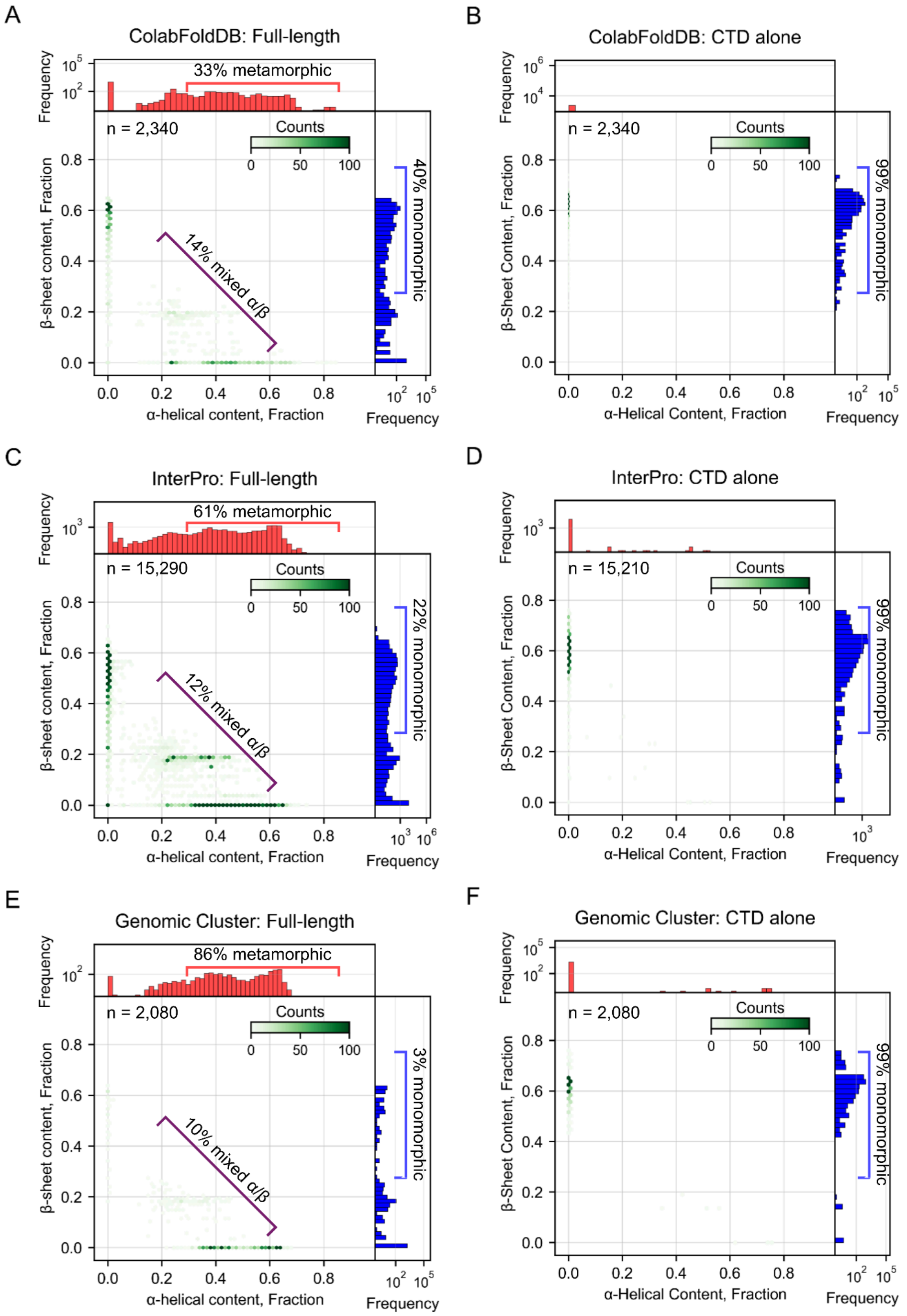

Supplementary Figure 2 Identification of metamorphic, mixed α/β CTD, and monomorphic RfaH homologs based on AF2 protein structure predictions. Secondary structure content of predictions of RfaH homologs from ColabFoldDB (**A**, **B**), InterPro (**C**, **D**), and a reported Genomic Cluster (**E**, **F**). Predictions were made using either the full-length RfaH sequences from each database (**A**, **C**, **E**) or the isolated CTD (**B**, **D**, **F**), determined based on MSAs generated using MAFFT. The secondary structure assignment of the CTD, determined using STRIDE, shows groups of predicted RfaH structures that display an αCTD, a βCTD, or mixed α/β secondary structure. Some structures from InterPro had short CTD sequences.

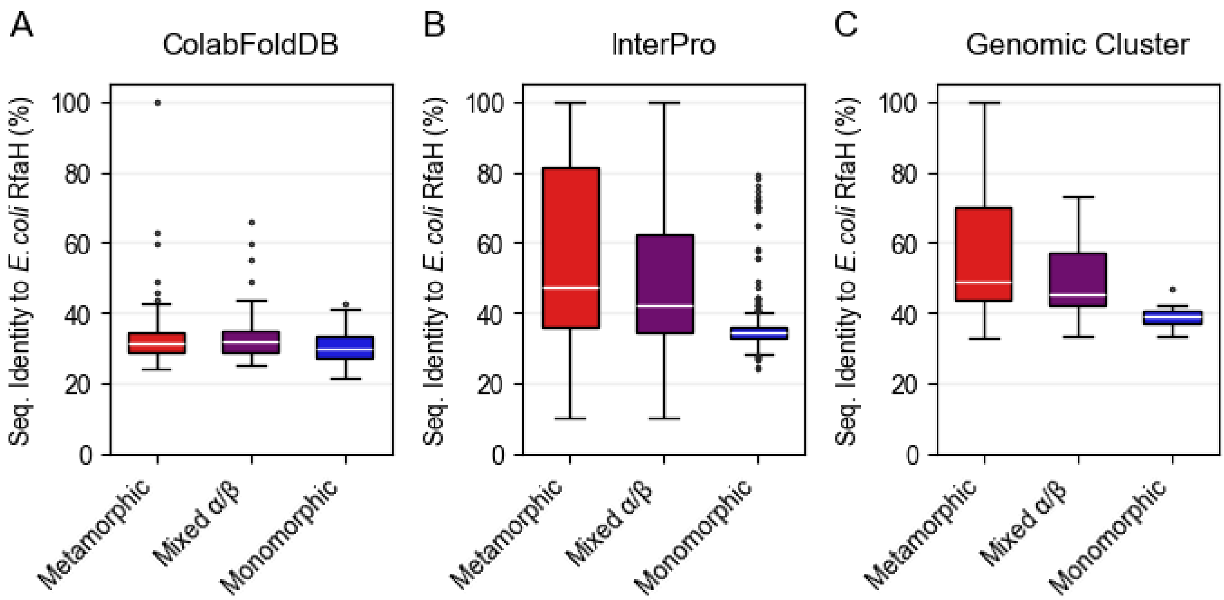

Supplementary Figure 3 Distribution of sequence identity against *E. coli* RfaH for all datasets. The structural predictions from the ColabFoldDB (**A**), InterPro (**B**) and (**C**) databases presented in Figure 2 were used to assign each structure to a CTD fold category, namely αCTD (red), βCTD (blue), or mixed α/β CTD (purple), and its sequence identity calculated against *E. coli* RfaH was appended to the corresponding category. Each sequence can be present at most once in every category, based on its predicted CTD. In the box plots, the median is shown as a horizontal white line, the lower and upper ends indicate the 25% and 75% interquartile range, and the whiskers extend to the percentile 1 and 99, with points representing outliers.

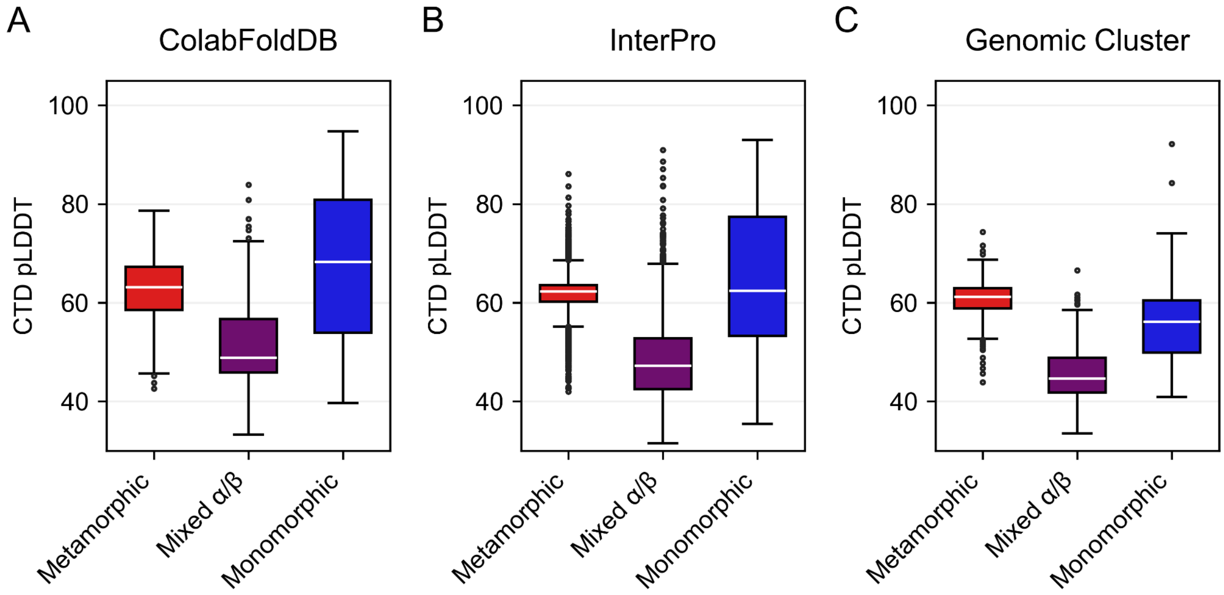

Supplementary Figure 4 Distribution of the pLDDT of the CTD of full-length RfaH protein structures from all databases. The average pLDDT of the CTD from the RfaH structures predicted form the ColabFoldDB (**A**), InterPro (**B**) and Genomic Cluster (**C**) datasets was used as a proxy to rank the best predicted structures, and these values are presented in these plots clustered by CTD fold category, namely αCTD (red), βCTD (blue), or mixed α/β CTD (purple). Each sequence can be present at most once in every category, based on its predicted CTD. In the box plots, the median is shown as a horizontal white line, the lower and upper ends indicate the 25% and 75% interquartile range, and the whiskers extend to the percentile 1 and 99, with points representing outliers.

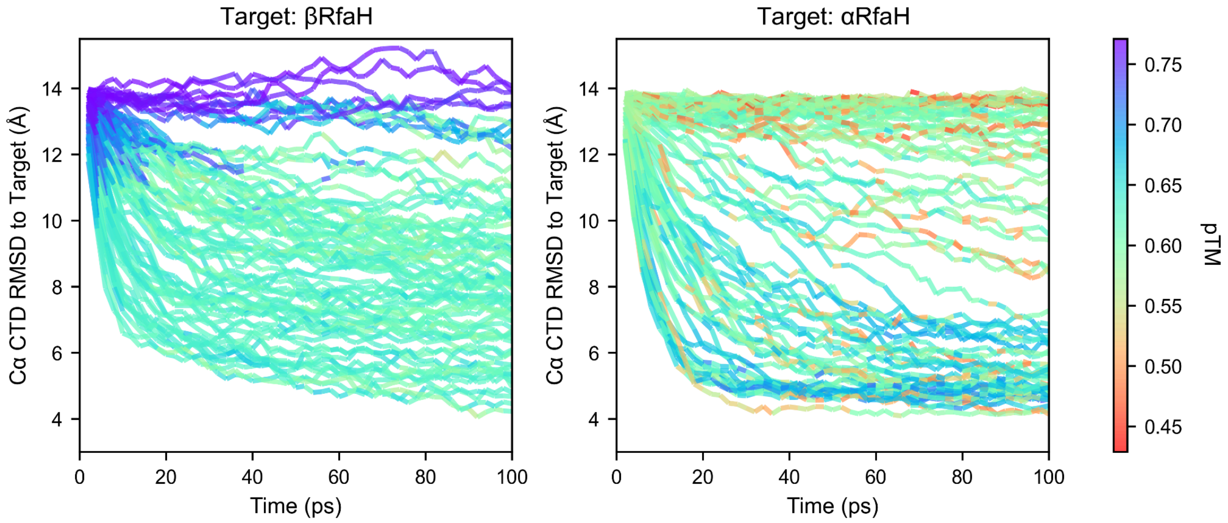

Supplementary Figure 5 Graphical representation of the TMD trajectories. A total of 200 TMD trajectories were produced, in which backbone atoms from residues 100-162, comprising *E. coli* RfaH CTD were subjected to an external potential to fold-switch from the autoinhibited (**A**) or the active state (**B**) towards the target opposite native state, and ~100 simulations were selected based on Principal Component Analysis to create thousands of decoys for further analysis using AF2Rank. Each line represents a single trajectory of 100 ps, with the colors indicating the predicted TM score (pTM) for each input structure. The plots show the Cα RMSD of *E. coli* RfaH CTD against the target structure, indicated in the title of each plot.

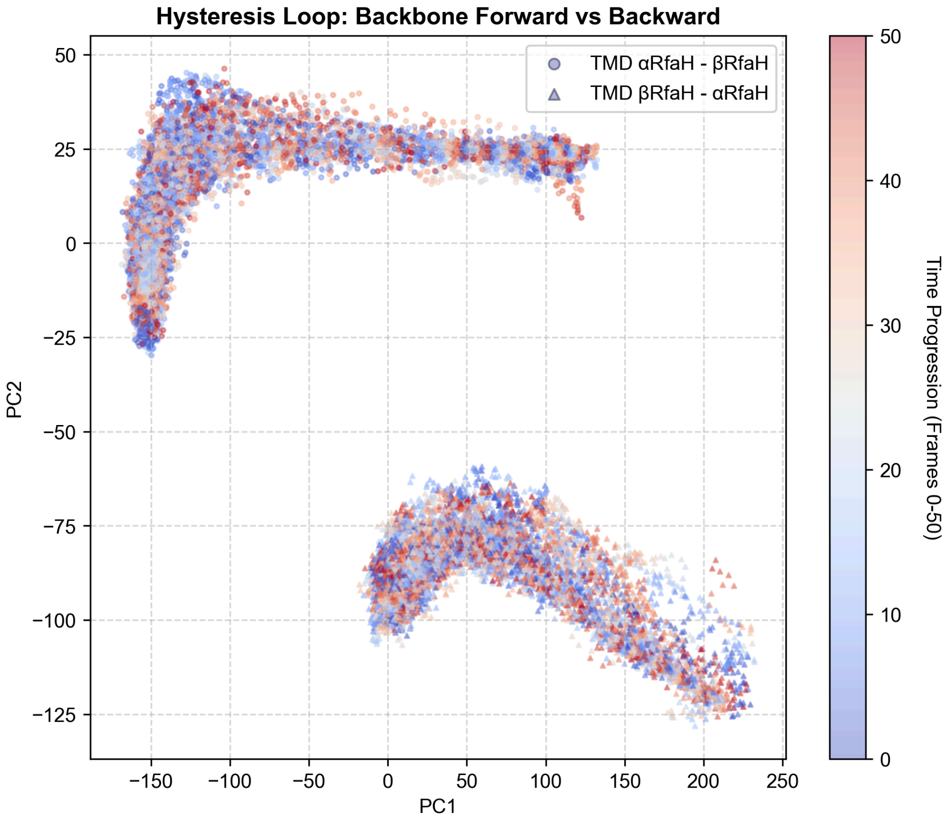

Supplementary Figure 6 Hysteresis of the TMD simulations. Principal Component Analysis (PCA) projections based on a coordinate covariance matrix, which was calculated using the Cα atoms of the CTD (residues 110–162) of *E. coli* RfaH, while the stable NTD (residues 1–100) was used as a rigid-body reference for alignment to remove translational and rotational motions. The covariance matrix was diagonalized to obtain the eigenvectors and project all 600 TMD trajectories (300 in each fold-switching direction) based on two components, PC1 and PC2, and the trajectory frames were clustered into at least 100 distinct groups based on their Euclidean distance in the PC1/PC2 subspace using *k*-means clustering. The centroid of each cluster was identified, and the specific trajectory containing the centroid frame was selected as a representative pathway. This process yielded a curated dataset of 100 simulations for the TMD trajectories from αRfaH to βRfaH and 99 trajectories from βRfaH to αRfaH.

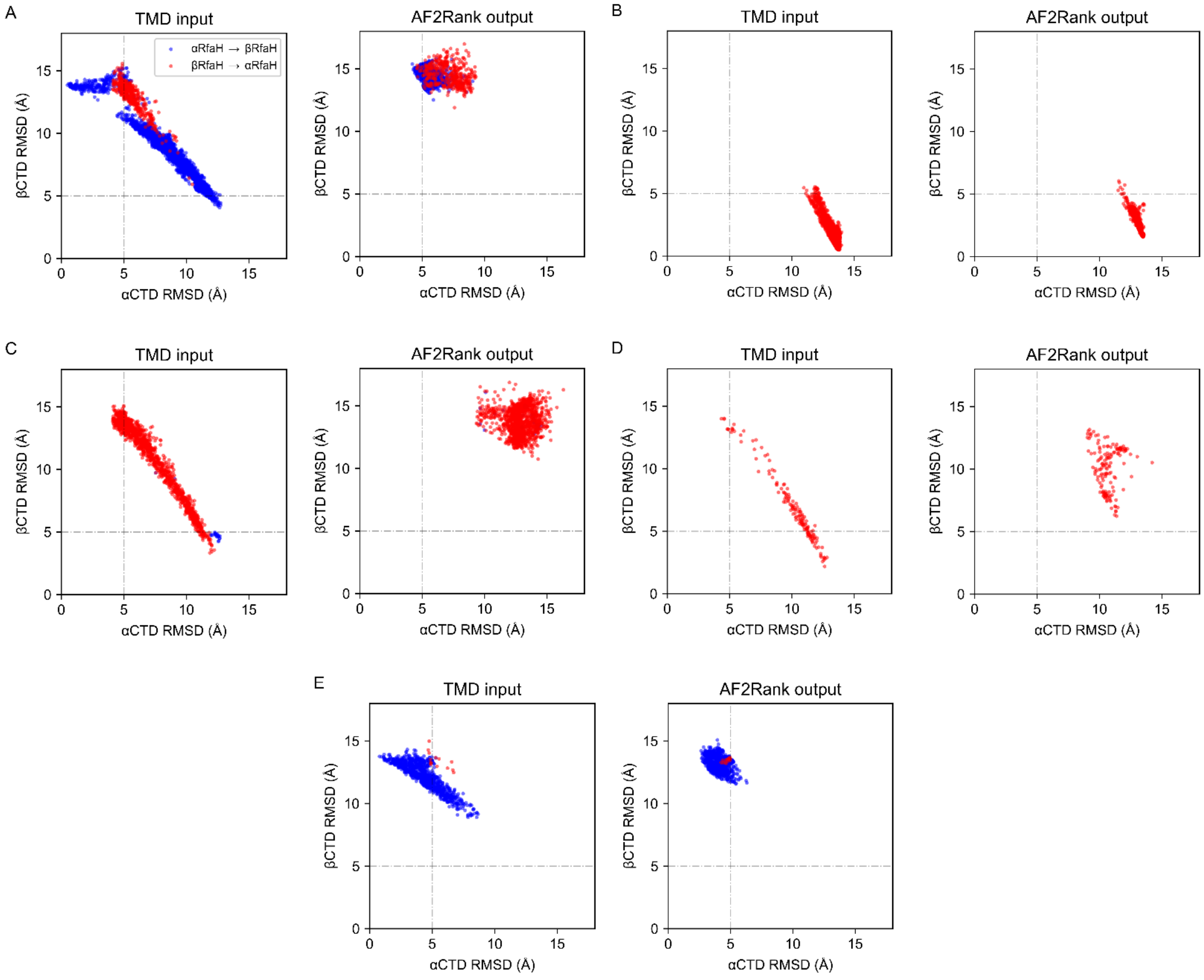

Supplementary Figure 7 Structural mapping of the input decoys generated by TMD and the output structures refined by AF2Rank. Cluster 1 (**A**) contains AF2Rank output structures generated from 2,471 decoys from TMD simulations from αRfaH to βRfaH and 472 decoys from the reverse simulations; cluster 2 (**B**) contains AF2Rank output structures from 1,704 input structures from TMD simulations from βRfaH to αRfaH; cluster 3 (**C**) contains AF2Rank output structures generated from 23 decoys from TMD simulations from αRfaH to βRfaH and 1,357 decoys from the reverse simulations; cluster 4 (**D**) contains AF2Rank output structures from 153 input structures from TMD simulations from βRfaH to αRfaH; and cluster 5 (**E**) contains AF2Rank output structures generated from 986 decoys from TMD simulations from αRfaH to βRfaH and 14 decoys from the reverse simulations. Clusters 1, 3 and 4 represent intermediate states with inverted helices (cluster 1), and mixed secondary structure (groups 3 and 4), as shown in Figure 4 in the main text, whereas clusters 2 and 5 resemble the active and autoinhibited states of *E. coli* RfaH, respectively.

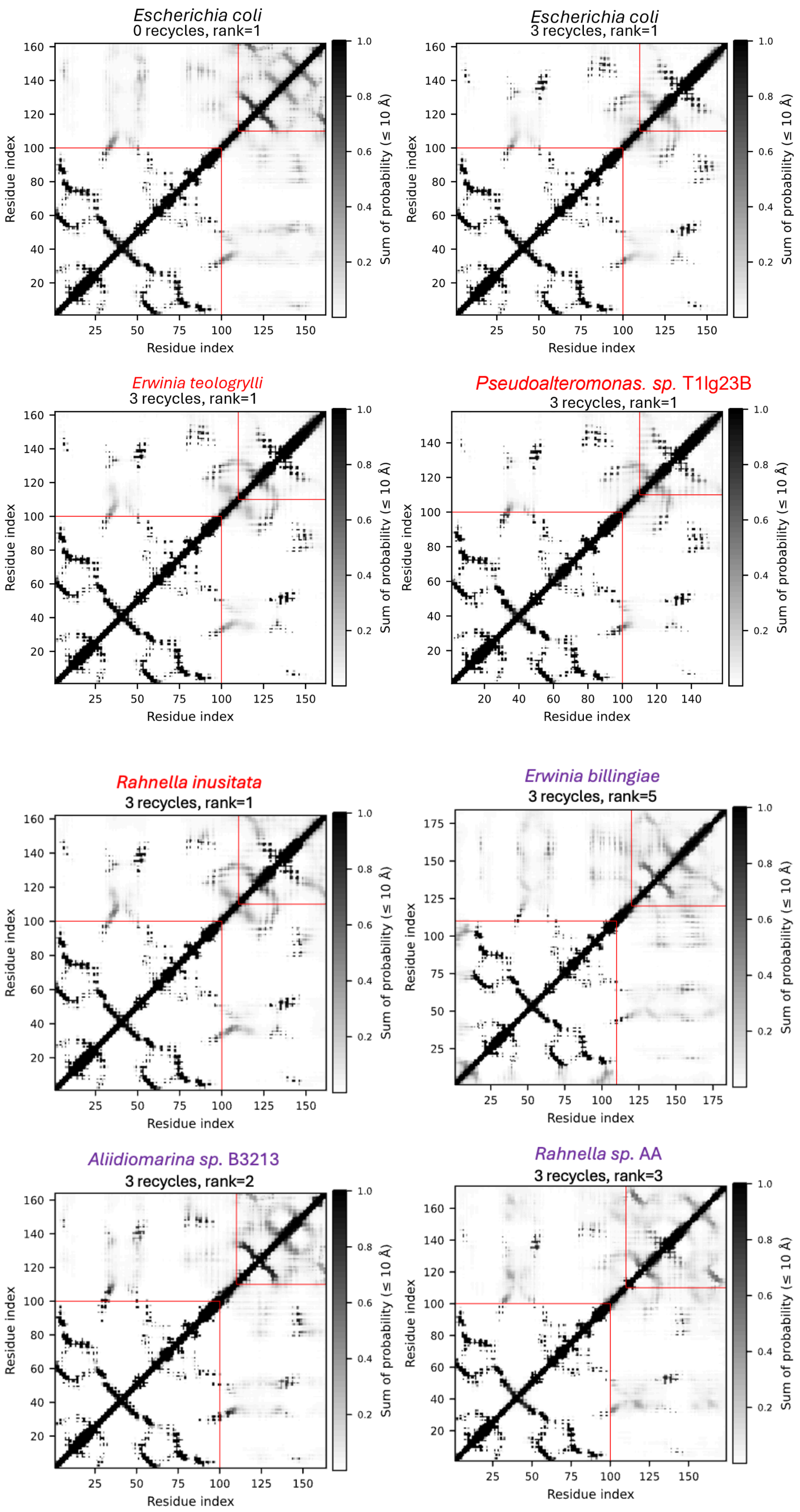

Escherichia coli
0 recycles, rank=1
Escherichia coli
3 recycles, rank=1
Erwinia teologrylli
3 recycles, rank=1
Pseudoalteromonas. sp. T1lg23B
3 recycles, rank=1
Rahnella inusitata
3 recycles, rank=1
Erwinia billingiae
3 recycles, rank=5
Aliidiomarina sp. B3213
3 recycles, rank=2
Rahnella sp. AA
3 recycles, rank=3
Residue index
Sum of probability (≤ 10 Å)

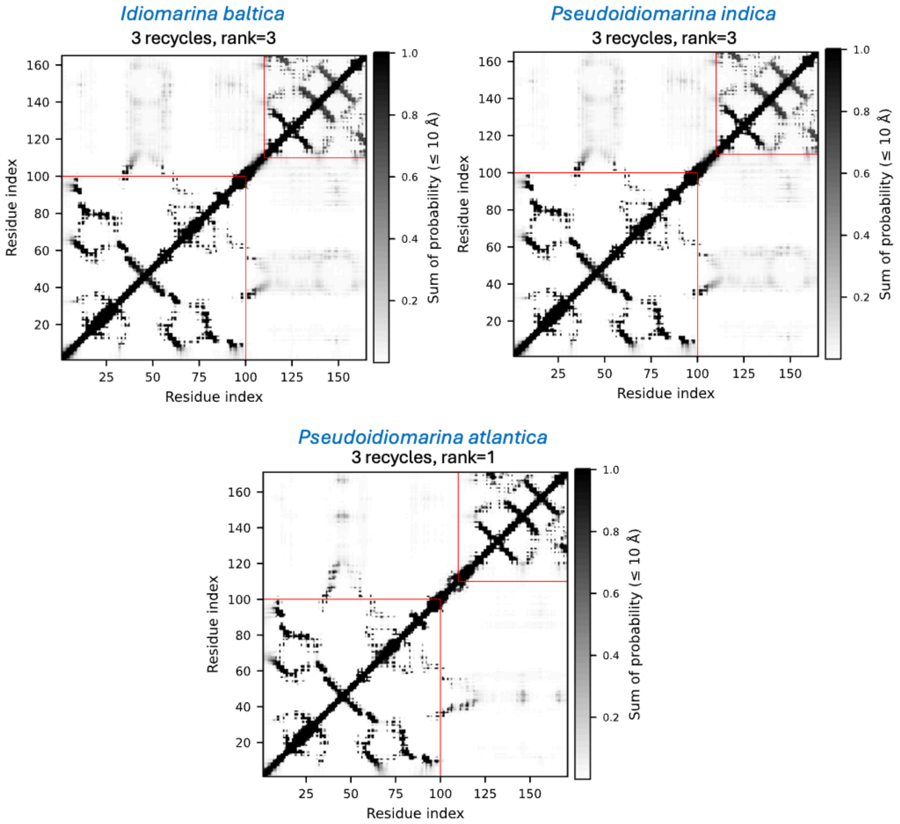

Supplementary Figure 8 Distograms for *E. coli* RfaH in the autoinhibited and active states and metamorphic, mixed α/β and monomorphic RfaH orthologs AF2 predictions from the Genomic Cluster. Distograms plot the contact probability up to 10 Å cutoff for RfaH orthologs predicted by AF2, highlighting the species name in the color code: *E. coli* (best ranked structures from ColabFold predictions at recycle 0 and recycle 3) in black, metamorphic orthologs in red, RfaH orthologs with mixed α/β CTD in purple, and RfaH homologs predicted as monomorphic in the canonical NusG-like active state in blue.

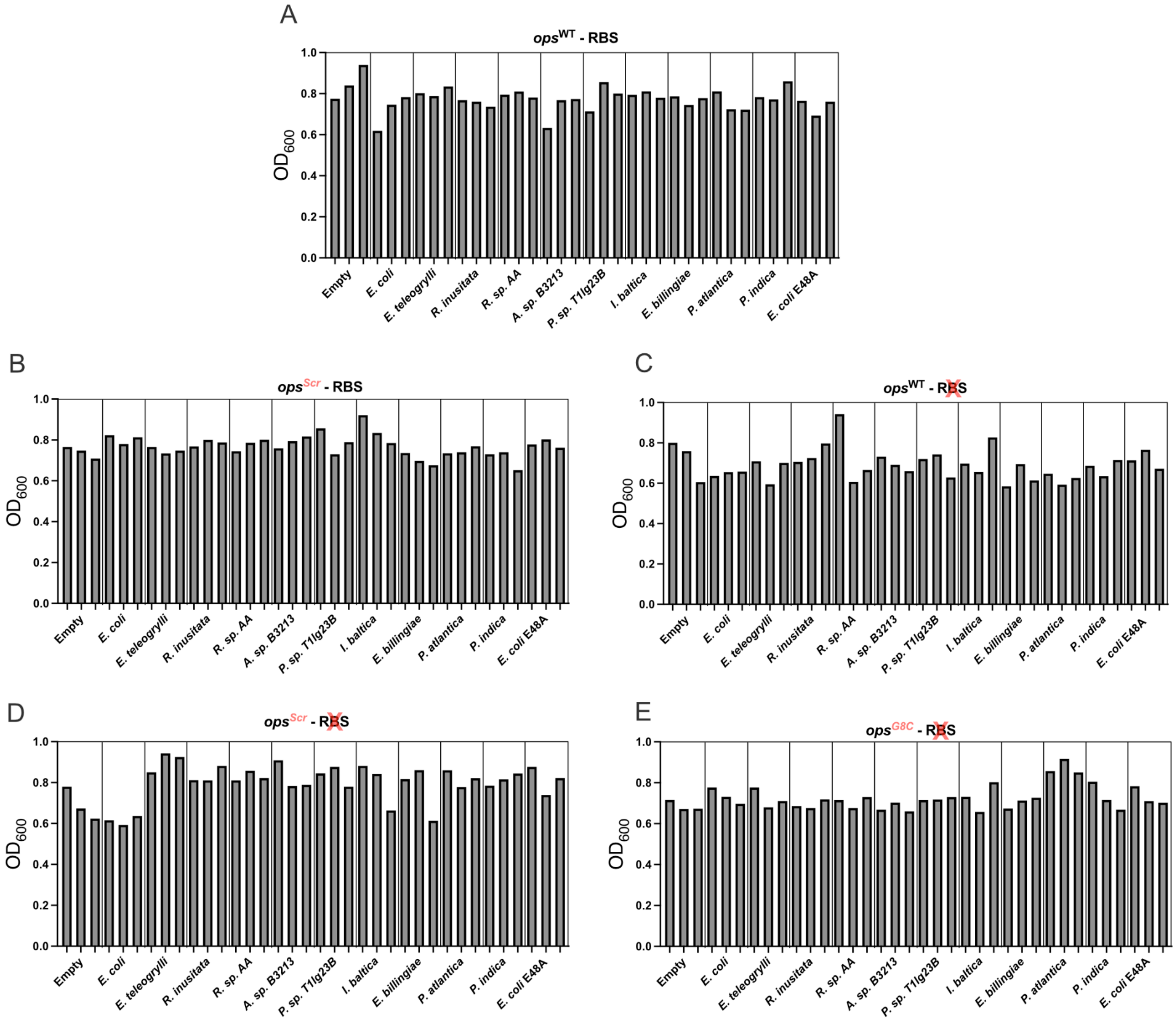

Supplementary Figure 9 Final OD$_{600}$ measured for each colony used in the *in vivo* luminescence assays under non-inducible conditions. The genetic architectures for the plasmid encoding the luminescence reporter correspond to *ops*^{WT} with RBS (**A**), *ops*^{Scr} with RBS (**B**), *ops*^{WT} without RBS (**C**), *ops*^{Scr} without RBS (**D**) and *ops*^{G8C} without RBS (**D**). Three biological replicates were performed per RfaH protein per condition.

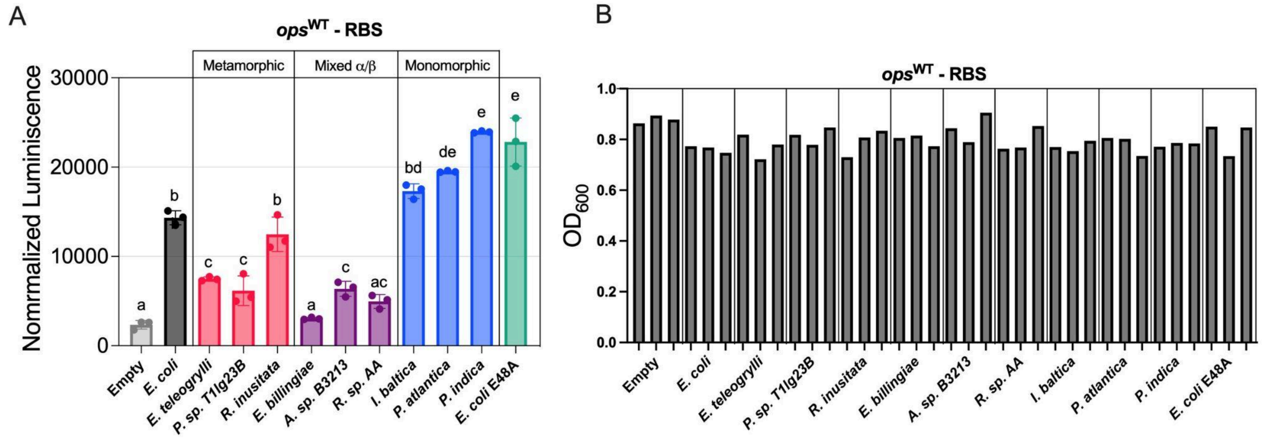

Supplementary Figure 10 *In vivo lux* assay under IPTG induction of expression of RfaH homologs. (**A**) The plasmid encoding the *luxCDABE* operon downstream of the wild-type *ops*-element and RBS was co-transformed with the plasmid encoding the RfaH-like proteins in *E. coli* DH5α Δ*rfaH* cells or with an empty vector as control. The *in vivo* luminescence assay was carried out using IPTG concentrations to induce RfaH overexpression (see *Methods*). Each bar represents the average raw luminescence level of six technical replicates of three colonies, normalised by cell density. The error bar shows the standard deviation. A one-way Analysis of Variance (ANOVA) was used to assess statistical significance, with all samples compared to each other. The same letter means non-significant different (p-value > 0.05), while different letter means statistical difference (p-value $\leq$ 0.05). (**B**) Final OD$_{600}$ measured for each colony used in the assay. The raw luminescence of each colony was normalized to its corresponding final OD^{600}. Three biological replicates were performed per RfaH protein per condition.

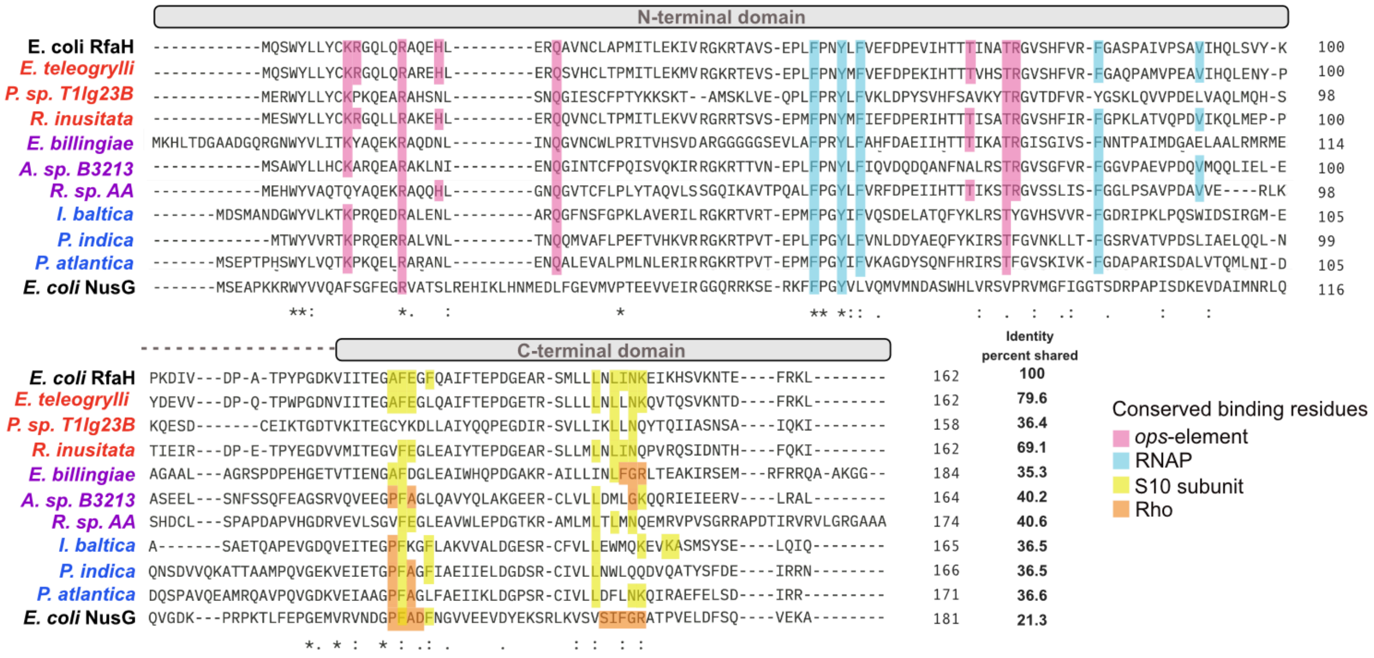

Supplementary Figure 11 MSA of RfaH orthologs tested in the *lux* assay. The MSA was generated using MUSCLE (Edgar, 2004) available at the EMBL-EBI Job Dispatcher web server (Madeira *et al*, 2024). The orthologs classified as metamorphic, mixed α/β CTD and monomorphic are labeled in red, purple, and blue, respectively. The MSA was built including the sequences of *E. coli* RfaH and NusG as references. The colors in the columns indicate the conservation of residues associated with binding to the *ops* DNA element (pink), RNAP (cyan), the S10 subunit (yellow) and Rho (orange). It can be observed that there is only partial conservation of *ops*-binding residues and of residues from loop 1 involved in Rho binding in the case of the monomorphic proteins.

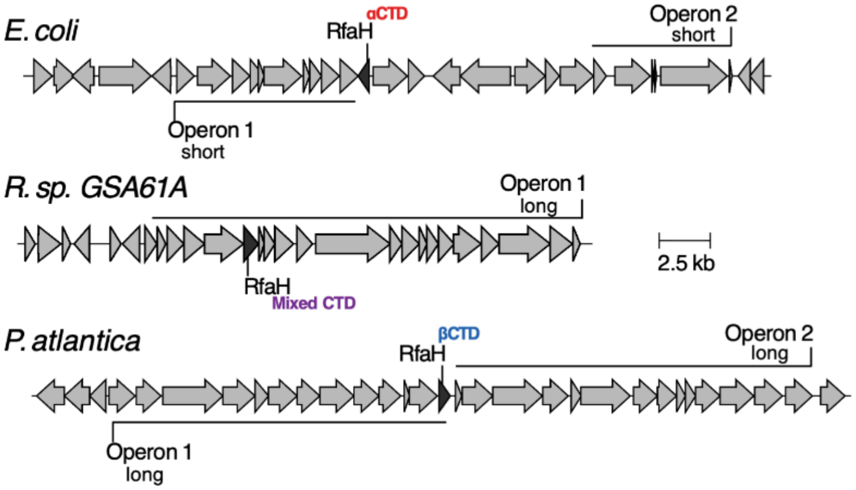

Supplementary Figure 12 Gene neighborhood of genomic-encoded RfaH orthologs. Representative genomic vicinity of the metamorphic, mixed α/β CTD and monomorphic RfaH experimentally ascertained in this work. The surrounding 15 genes up and downstream of the *rfaH* gene are shown, as well as their open reading frame (ORF) direction, for three organisms: *E. coli* RfaH, the canonical metamorphic protein; *Rhanella sp.* RfaH, belonging to the mixed α/β CTD clade; and *P. atlantica* RfaH, belonging to the monomorphic clade. Based on the operon definition described in the *Methods* section, different operon lengths near or away from the RfaH production site can be observed among these three examples, with the mixed α/β CTD of *Rhanella sp.* and the monomorphic RfaH from *P. atlantica* being part of an operon and acting in *cis*, whereas *E. coli* contains operons for which RfaH must act in *trans*.

SUPPLEMENTARY TABLES

Supplementary Table 1 Summary of selected RfaH proteins for *in vivo* luminescence assay.

Organism	AF2 Prediction	Topology	Sequence identity against *E. coli* RfaH
Escherichia coli	5α 0α/β 0β	Metamorphic	100%
Erwinia teleogrylli	5α 0α/β 0β	Metamorphic	79.6%
Pseudoalteromonas. sp. T1lg23B	5α 0α/β 0β	Metamorphic	36.4%
Rahnella inusitata	3α 2α/β 0β	Metamorphic	69.1%
Erwinia billingiae	1α 2α/β 2β	Mixed α/β	35.3%
Aliidiomarina sp. B3213	2α 2α/β 1β	Mixed α/β	40.2%
Rahnella sp. AA	1α 4α/β 0β	Mixed α/β	40.6%
Idiomarina baltica	2α 1α/β 2β	Monomorphic	36.5%
Pseudidiomarina indica	1α 0α/β 4β	Monomorphic	36.5%
Pseudidiomarina atlantica	0α 0α/β 5β	Monomorphic	36.6%

Supplementary Table 2 Plasmids used in this work

Plasmids	Name	Description	Resistance	Reference
ops and RBS variants for *lux* assay	pIA955	P_{BAD}–*ops*^{WT}_RBS-*luxCDABE*	Amp	(Belogurov *et al*, 2010)
	pIA1087	P_{BAD}–*ops*^{WT}–*luxCDABE*	Amp	(Burmann *et al*, 2012)
	pZL23	P_{BAD}–*ops*^{G8C}–*luxCDABE*	Amp	(Zuber *et al*, 2018)
	pZL4	P_{BAD}–*ops*^{Scr}_RBS-*luxCDABE*	Amp	(Zuber *et al*, 2018)
	pZL8	P_{BAD}–*ops*^{Scr}–*luxCDABE*	Amp	(Zuber *et al*, 2018)
Expression vector for *lux* assay	pIA947	P_{trc}- no CDS (“Empty”)	Cm	(Belogurov *et al*, 2010)
	pIA957	P_{trc}- RfaH$^{E. coli}$	Cm	(Belogurov *et al*, 2010)
	pIA1072	P_{trc}- RfaH$^{E. coli}$ (E48A)	Cm	(Burmann *et al*, 2012)
	pDP10	P_{trc}- RfaH$^{E. teologrylli}$	Cm	This work
	pDP11	P_{trc}- RfaH$^{R. inusitata}$	Cm	This work
	pDP12	P_{trc}- RfaH$^{R. sp. AA}$	Cm	This work
	pDP13	P_{trc}- RfaH$^{A. sp. B3213}$	Cm	This work
	pDP14	P_{trc}- RfaH$^{P. sp. T1lg23B}$	Cm	This work
	pDP15	P_{trc}- RfaH$^{Idiomarina\ baltica}$	Cm	This work
	pDP16	P_{trc}- RfaH$^{E. billingiae}$	Cm	This work
	pDP17	P_{trc}- RfaH$^{P. atlantica}$	Cm	This work
	pDP18	P_{trc}- RfaH$^{P. indica}$	Cm	This work

SUPPLEMENTARY REFERENCES

Belogurov GA, Sevostyanova A, Svetlov V & Artsimovitch I (2010) Functional regions of the N-terminal domain of the antiterminator RfaH. *Mol Microbiol* 76: 286–301

Burmann BM, Knauer SH, Sevostyanova A, Schweimer K, Mooney RA, Landick R, Artsimovitch I & Rösch P (2012) An α helix to β barrel domain switch transforms the transcription factor RfaH into a translation factor. *Cell* 150: 291–303

Edgar RC (2004) MUSCLE: multiple sequence alignment with high accuracy and high throughput. *Nucleic Acids Res* 32: 1792–1797

Madeira F, Madhusoodanan N, Lee J, Eusebi A, Niewielska A, Tivey ARN, Lopez R & Butcher S (2024) The EMBL-EBI Job Dispatcher sequence analysis tools framework in 2024. *Nucleic Acids Res* 52: W521–W525

Zuber PK, Artsimovitch I, NandyMazumdar M, Liu Z, Nedialkov Y, Schweimer K, Rösch P & Knauer SH (2018) The universally-conserved transcription factor RfaH is recruited to a hairpin structure of the non-template DNA strand. *Elife* 7: e36349
